# Correction: Foraging as sampling without replacement: A Bayesian statistical model for estimating biases in target selection

**DOI:** 10.1371/journal.pcbi.1010997

**Published:** 2023-03-17

**Authors:** Alasdair D. F. Clarke, Amelia R. Hunt, Anna E. Hughes

The data in Fig 9 contains errors related to the analysis code. In the two upper plots, the model’s run length statistics were merged with the data from the human participants. As a result, the data labelled “empirical run statistic” was an average of the human and model data, leading to overly strong correlations with the “predicted run statistics”. In addition, during the computation of the inter-target distances, an extra x was used in place of a y. The corrected plots in the lower half of the figure show a closer agreement between model and human participants than the original. The authors have provided a corrected Fig 9 here.

In the last paragraph of the Results section, sentences 4–10 should be removed. The complete paragraph should read:

Finally, we can use the fitted model to simulate new data and compare run statistics and inter-target distances with the original empirical data. We do this for n = 100 samples from the fitted posterior probability distributions so that we can quantify the uncertainty in the model’s predictions. The results (Fig 9) show a good correspondence between the posterior predictions and empirical data.

**Fig 9:**
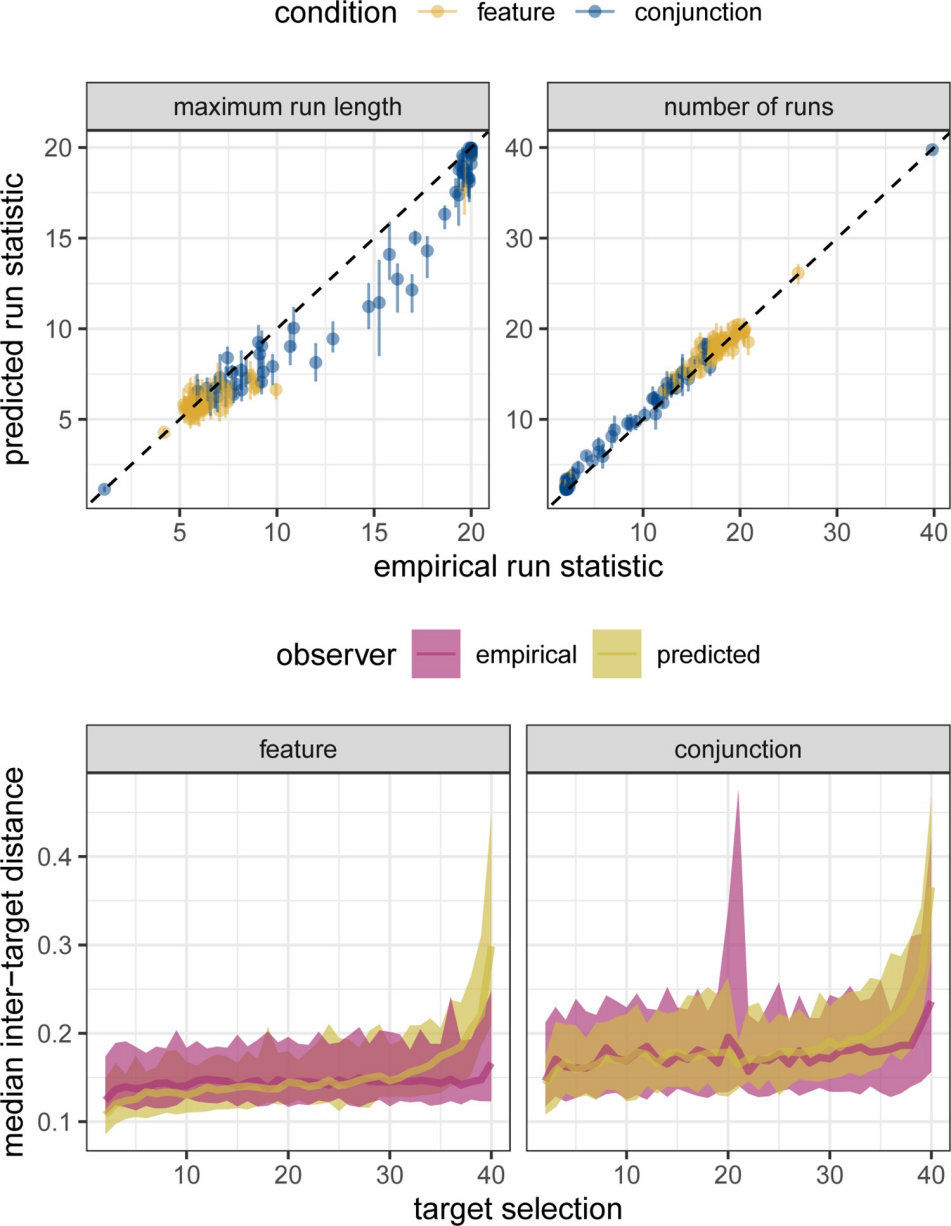
Posterior predictions for Clarke et al. (2018). (*top*:) Scatter plots between empirical and predicted summary run statistics. Error bars indicated 89% HPDI for the posterior predictions. (*bottom*:) Comparison of inter-target distances and how they vary over time. Shaded region gives 89% intervals.
