# Supplementary material for: Foraging as sampling without replacement: A Bayesian statistical model for estimating biases in target selection
Source: PLoS Comput Biol. 2022 Jan 24;18(1):e1009813. doi: 10.1371/journal.pcbi.1009813 (PMC8812991; doi:10.1371/journal.pcbi.1009813)
Supplement: S1 File — Supplementary materials for the bag foraging model. (PDF) [file pcbi.1009813.s001.pdf]

Foraging for a Model

A Clarke and A Hughes

05/03/2021

Foraging in chickens: Dawkins (1971)

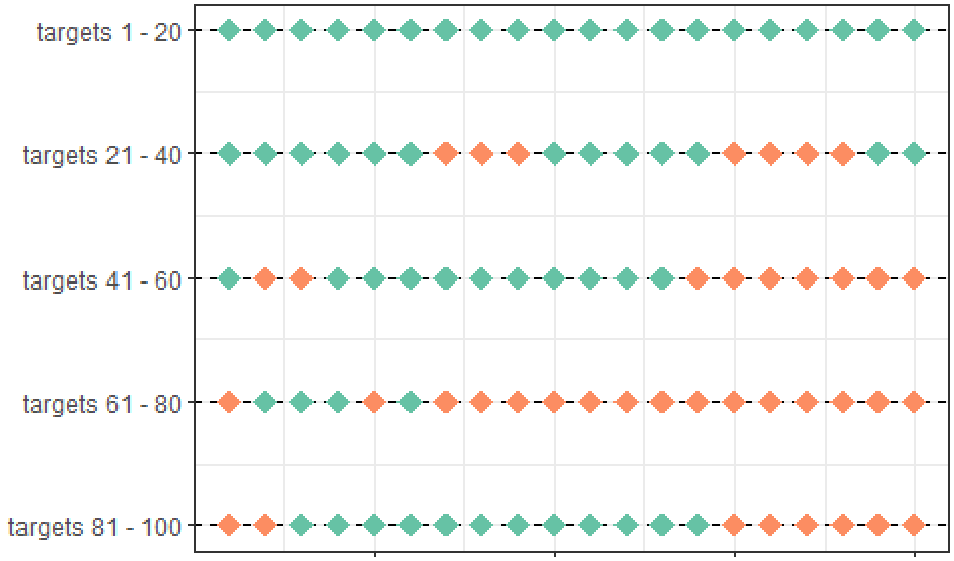

Fig 1: *Re-creating Figure 2 of Dawkins (1971): the first 100 grains taken by one chick in experiment 1.*

[1] is often referenced as one of the classic papers in the foraging literature. In experiment 1 of this paper, five chicks were given two types of food ('conspicuous' green or 'cryptic' orange grains of rice, 1000 grains of each) and the order in which they ate the rice grains was recorded. The key finding was that the chicks ate the first 100 grains in 'runs', repeatedly selecting one colour and then another. Dawkins argued that the pattern of selection differed from what would be expected from random chance (using a one sample runs test for each chick), suggesting that chicks switch their attention during feeding.

Bayesian model fitting

We fit a Bayesian model to the first 100 trials of one of the chicks in [1]. Our model takes into account the total number of grains that were presented to the chick (2000) and attempts to calculate p_s (whether the chick had a preference to stick with one grain type: $p_s = 0.5$ indicates random switching, a higher value indicates a stronger tendency towards 'runs' of one colour). Note that $b_s$in the table and figures below is the untransformed parameter from the model fit: to generate p_s, we simply take the inverse logistic of b_s.

Table 1: *Model summary.*

	mean	se_mean	sd	2.5%	25%	50%	75%	98%	n_eff	Rhat
bS	1.49	0.01	0.24	1.03	1.33	1.48	1.65	1.96	1596	1
pS	0.81	0.00	0.04	0.74	0.79	0.81	0.84	0.88	1609	1
b_prior	0.01	0.01	0.50	-0.99	-0.31	0.03	0.35	0.97	3798	1
p_prior	0.50	0.00	0.12	0.27	0.42	0.51	0.59	0.73	3782	1
lp__	-41.55	0.02	0.70	-43.55	-41.73	-41.27	-41.09	-41.04	1865	1

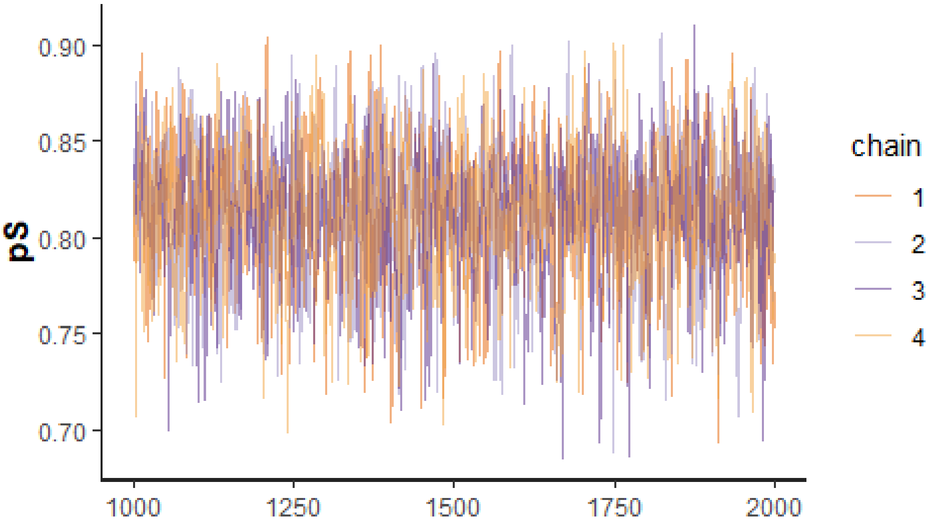

Fig 2: *Trace plot for Bayesian model fit on Dawkins (1971) data. The x axis indicates the sample number (2000 iterations were used per chain in the model).*

The traceplot suggests the model has fit well (all chains are well mixed).

Posterior

Table 2: *HDCI intervals for the posterior distribution of p_S.*

width	lower	upper
0.53	0.80	0.85
0.97	0.73	0.88

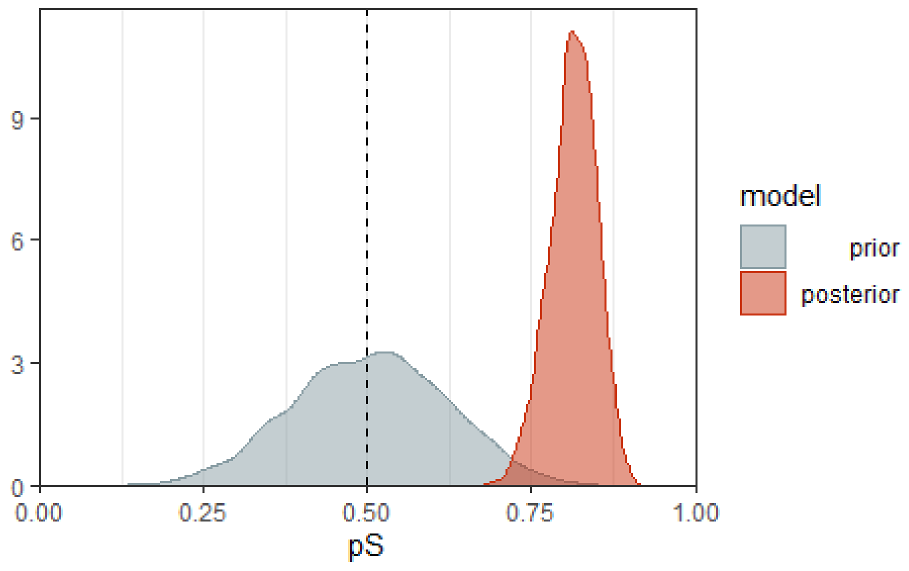

Fig 3: *Prior and posterior probability distributions for* p_s*.*

In agreement with the original conclusions of Dawkins (1971) we see evidence for chicks having a bias towards longer "runs" of one grain colour, rather than random selection.

Posterior Predictions

We can evaluate our model by calculating posterior predictions and comparing them to the original data.

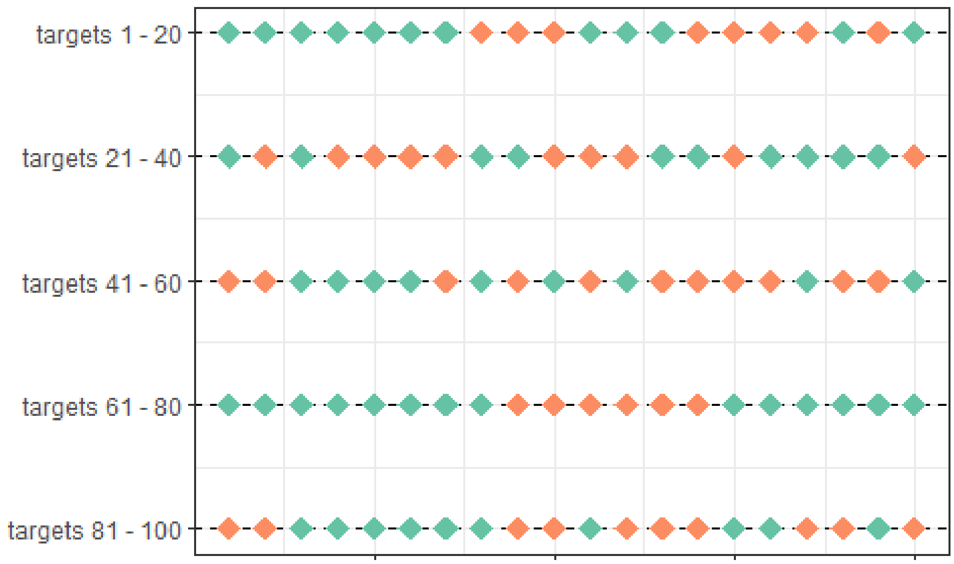

Fig 4: *Simulated Chicken.*

We can now repeat this process for a load of samples from our posterior and calculate the expected distribution of maximum run lengths and number of runs.

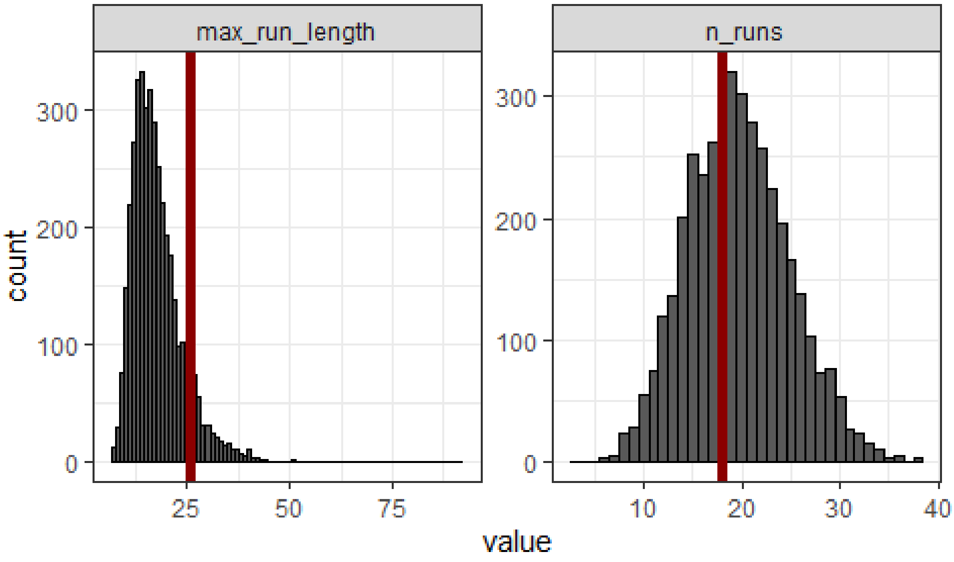

Fig 5: *Comparing our flock of simulated chickens (grey) to the real chicken (red).*

Sensitivity Analysis: Number of targets and length of trial

Dawkins' chicken pecked 100 / 2000 grains. What happens if we simulate the experiment with the chickens pecking a greater proportion of the grains?

We ran a simulation where the chickens either found (i) 100 grains (ii) 200 grains or (iii) 2000 grains (out of a total of 2000 grains).

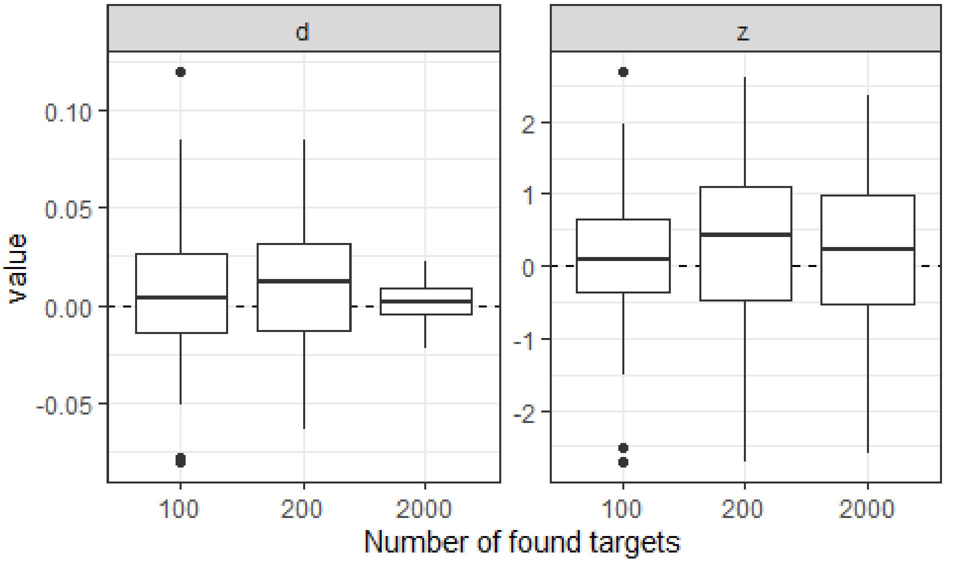

Fig 6: *Left hand panel: d represents the difference between the simulated value and the ground truth (taken to be* $p_s = 0.8$*). Right hand panel: z is the difference divided by the standard error (σ).*

We also ran a simulation where the chickens always found 100 grains, but the total number of grains varied, with three possible totals: (i) 100 grains (ii) 200 grains or (iii) 2000 grains.

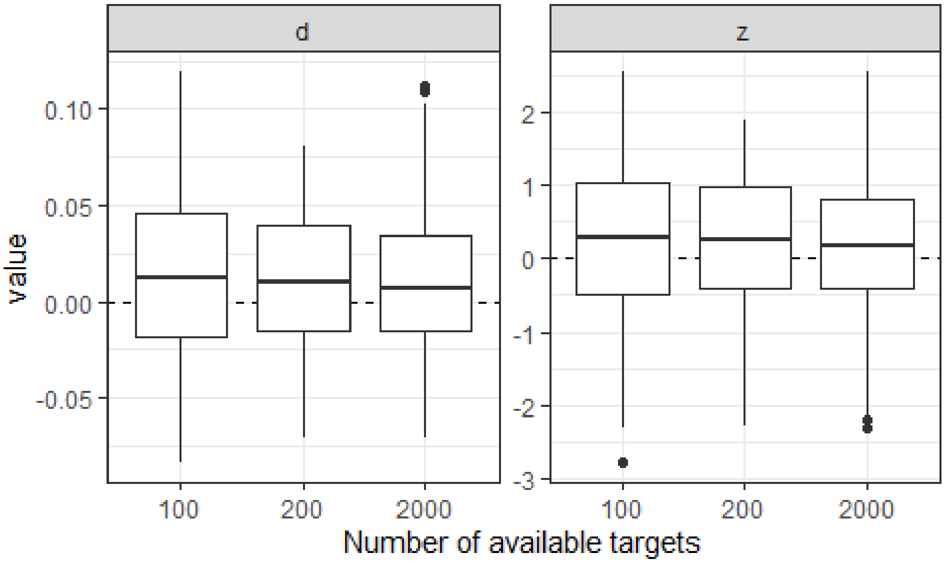

Fig 7: *Left hand panel: d represents the difference between the simulated value and the ground truth (taken to be* p_s *= 0.8). Right hand panel: z is the difference divided by the standard error (σ).*

In both cases, we can see that our model is typically pretty good, but slightly underestimates the ground truth on average when the chicken is pecking at only a small number of targets, regardless of whether this represents a small proportion of the total targets or the entire set of targets (d is calculated as the ground truth value minus μ, thus a positive value indicates μ is smaller than the ground truth). More targets are indeed better, although the difference is perhaps less obvious when normalised by the standard error.

Simulation examples: Multiple trials & conditions

Extending to multiple trials and conditions allows us to introduce a new parameter: p_a, the probability an observer prefers target type A.

Example: unbiased

Initially, we will give examples where our simulated observers are unbiased with respect to both p_a and p_s:

```
prob_a <- 0.5 # p(x=a | equal numbers of a and b balls left)
prob_s <- 0.5 # p(x_i = x_{i-1} | equal numbers of a and b balls left)
```

Summary statistics for a large dataset

First, we will give the distributions over 10k trials. These show the baseline/null distributions.

We can see that the number of runs is 21 (slightly over $n_t/2$) and the maximum run length is approximately 5, though the distribution is slightly skewed.

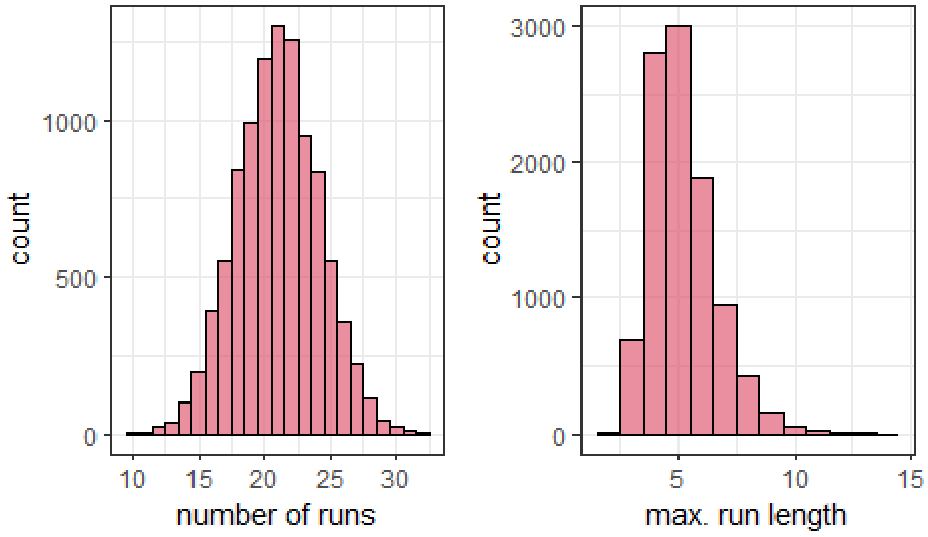

Fig 8: *Left hand panel: distribution of number of runs in our simulated large dataset. Right hand panel: distribution of maximum run lengths in our simulated large dataset.*

Table 3: *Summary statistics for our large dataset*

feature	mean	sd
numruns	21.0	3.1
maxrun	5.2	1.4

A smaller sample size

Now, we will use a smaller dataset. We will use the values below for the amount of data, based on the typical sample sizes used in similar studies. In the examples below, a simulated observer carries out a number of trials, each with a number of targets. There is no early trial termination. p_a and p_s are both 0.5.

```
n_obs <- 1 # number of observers
n_trials <- 50 # number of trials per observer
n_targ <- 40 # the number of targets to find per trial

sigma_prior <- 0.5
```

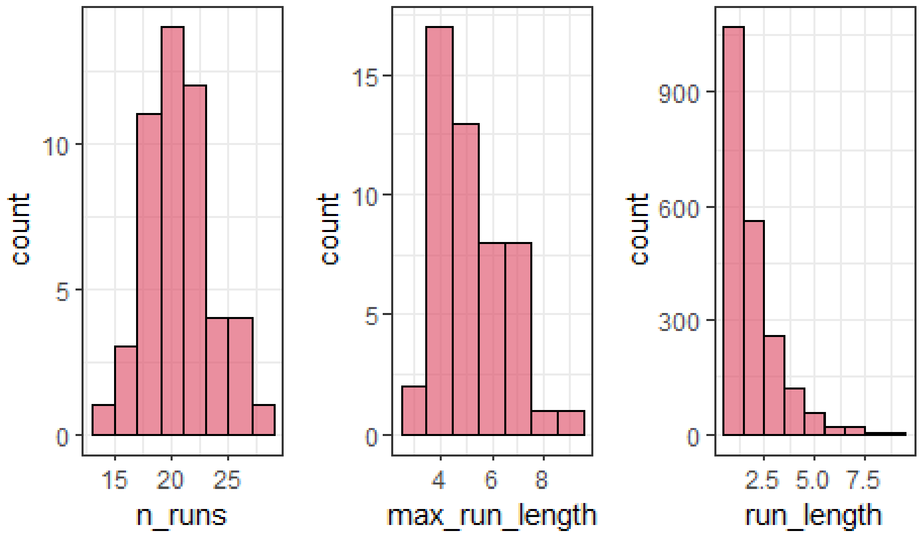

Fig 9: *Left: counts of number of runs. Middle: counts of maximum run length. Right: density plot of run length.*

Table 4: *summary statistics for our smaller dataset*

feature	mean	sd
numruns	21.1	3.0
maxrun	5.2	1.3

The summary graphs show some derived metrics that are commonly used in foraging studies. As can be seen, the value of e.g. the maximum run length can vary quite considerably even for unbiased trials.

Bayesian model fitting

We now fit our Bayesian 'bag foraging' model to the small dataset.

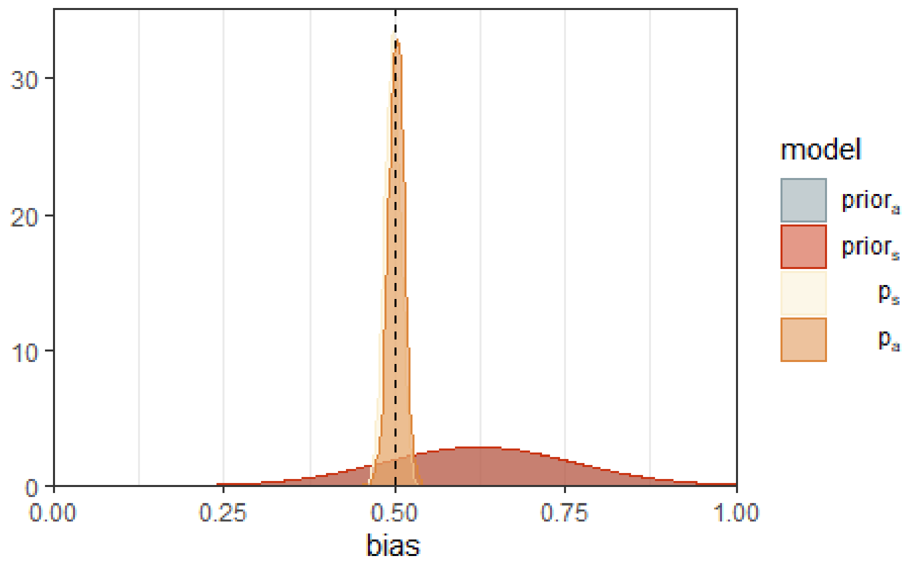

Fig 10: *Posterior plot for unbiased example. Note that* $prior_s$ *and* $prior_a$ *are identical, and thus their distributions entirely overlap.*

Table 5: *Model parameters for unbiased example.*

	mean	se_mean	sd	2.5%	25%	50%	75%	98%	n_eff	Rhat
bA[1]	0.01	0.00	0.05	-0.08	-0.02	0.01	0.04	0.10	964.7	1
bS[1]	-0.01	0.00	0.05	-0.10	-0.05	-0.01	0.02	0.08	969.1	1
pA[1]	0.50	0.00	0.01	0.48	0.49	0.50	0.51	0.52	964.5	1
pS[1]	0.50	0.00	0.01	0.47	0.49	0.50	0.50	0.52	969.2	1
bA_prior	0.00	0.02	0.49	-0.92	-0.32	0.00	0.33	0.96	943.9	1
bS_prior	0.02	0.02	0.50	-0.94	-0.33	0.02	0.36	0.99	923.4	1
pA_prior	0.62	0.00	0.00	0.62	0.62	0.62	0.62	0.62	0.5	1
pS_prior	0.62	0.00	0.00	0.62	0.62	0.62	0.62	0.62	0.5	1
lp__	-1283.40	0.05	0.98	-1286.17	-1283.74	-1283.10	-1282.72	-1282.46	470.4	1

Our model is able to recover both p_a and p_s accurately.

Example: small biases

We will now consider two conditions, one null, the other with a small bias (either in p_a or p_s).

A small bias to stay

First, we consider the case where an observer is slightly more likely to stay with the same type of target as the previous trial i.e. $p_s > 0.5$.

```
prob_a <- c(0.5, 0.5) # p(x=a | equal numbers of a and b balls left)
prob_s <- c(0.5, 0.6) # p(x_i = x_{i-1) | equal numbers of a and b balls left
)
```

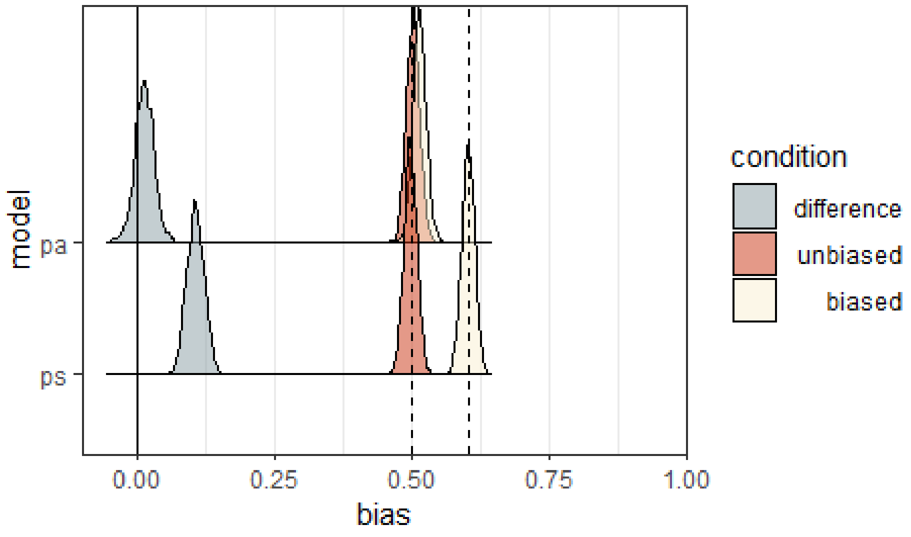

Fig 11: *Posterior plots for the case with a small bias in* p_s*.*

Table 6: *Probability that the difference between the model parameters is greater than zero.*

param	p_diff_greater_zero
pa	0.76
ps	1.00

The model is able to detect the small bias in p_s while recovering the unbiased parameter for p_a).

A small bias in target preference

Second, we consider the case where the observer has a slight preference for target A i.e. $p_a > 0.5$.

```
prob_a <- c(0.5, 0.6) # p(x=a | equal numbers of a and b balls left)
prob_s <- c(0.5, 0.5) # p(x_i = x_{i-1} | equal numbers of a and b balls left
)
```

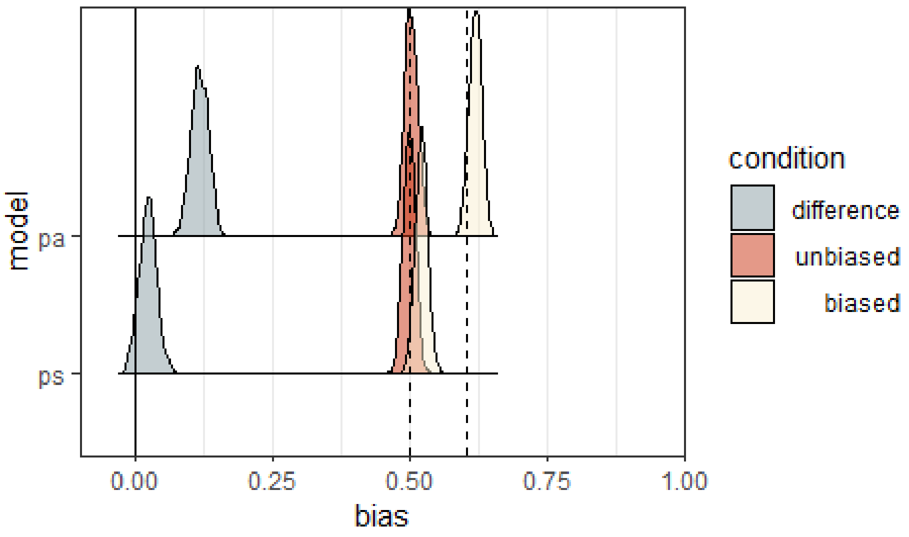

Fig 12: *Posterior plots for the case with a small bias in $p(a)$.*

Table 7: *Probability that the difference between the model parameters is greater than zero.*

param	p_diff_greater_zero
pa	1.00
ps	0.91

Again, the model is able to distinguish the biased parameter (p_a in this case) from the unbiased (p_s).

Example: large biases

Extreme

One possible large bias is that an observer could select all the targets of one type, and then proceed to the other target type i.e. they could have a $p_s = 1$. The opposite is also possible: the observer may select all of target type A first, and only then proceed onto target type B i.e. $p_a = 1$.

```
prob_a <- c(0.5, 1.0) # p(x=a | equal numbers of a and b balls left)
prob_s <- c(1.0, 0.5) # p(x_i = x_{i-1} | equal numbers of a and b balls left
)
```

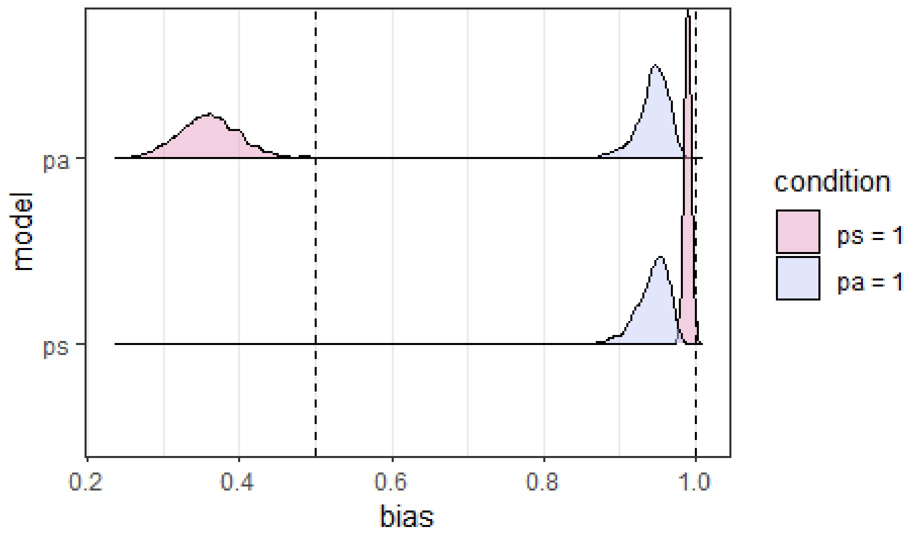

Fig 13: *Posterior plots for example with large p_s and large p_a biases.*

The model is able to find the correct value for p_s when its bias is high, but it struggles with p_a: the posterior does not include the true value 0.5. Similarly, when p_a is high, p_s is (incorrectly) high as well: although in this case, it is indeed difficult to specify p_s, as a very strong preference for always picking one target type before the other will look indistinguishable from always sticking rather than switching. (Note that this does not hold the other way round - a person can have a very strong preference to stick i.e. $p_s = 1$, but can show no preference for which target type they start with i.e. $p_a = 0.5$).

A little less extreme: p_a = 0.97

Arguably, in the real world, the extreme values of $p = 1$ are unlikely to occur. Therefore, we also tested the case where $p_a = 0.97$.

```
prob_a <- c(0.5, 0.97) # p(x=a | equal numbers of a and b balls left)
prob_s <- c(0.97, 0.5) # p(x_i = x_{i-1) | equal numbers of a and b balls lef
t)
```

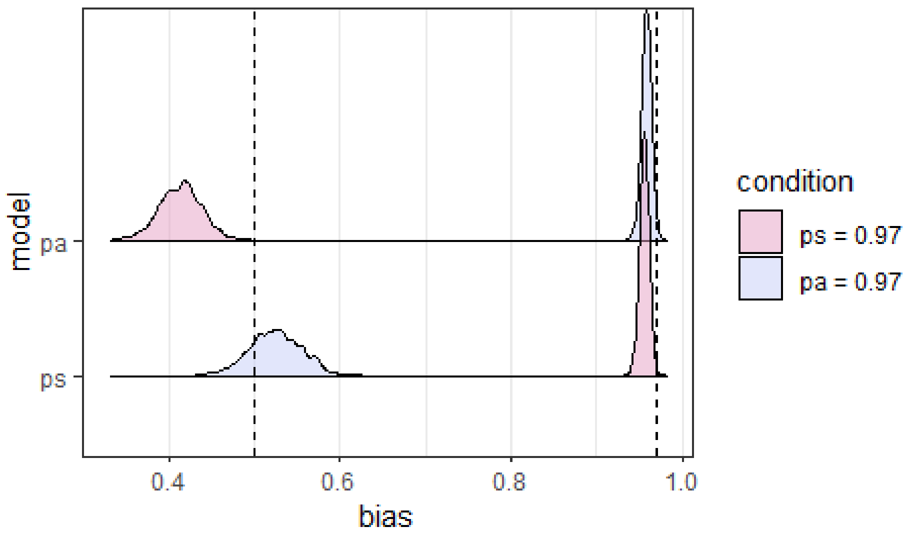

Fig 14: *Posterior plots for example with p_s and large p_a biases of 0.97.*

This model is better able to recover the correct parameters, although there is still a correlation between p_a and p_s.

Adjusting the prior

Another option would be to set a narrower prior (although note that this doesn't necessarily recover perfect parameters still, and may require very strong assumptions about the parameter values).

```
prob_a <- c(0.5, 1.0) # p(x=a | equal numbers of a and b balls left)
prob_s <- c(1.0, 0.5) # p(x_i = x_{i-1) | equal numbers of a and b balls left
)

sigma_prior_s <- 0.05
sigma_prior_a <- 0.5
```

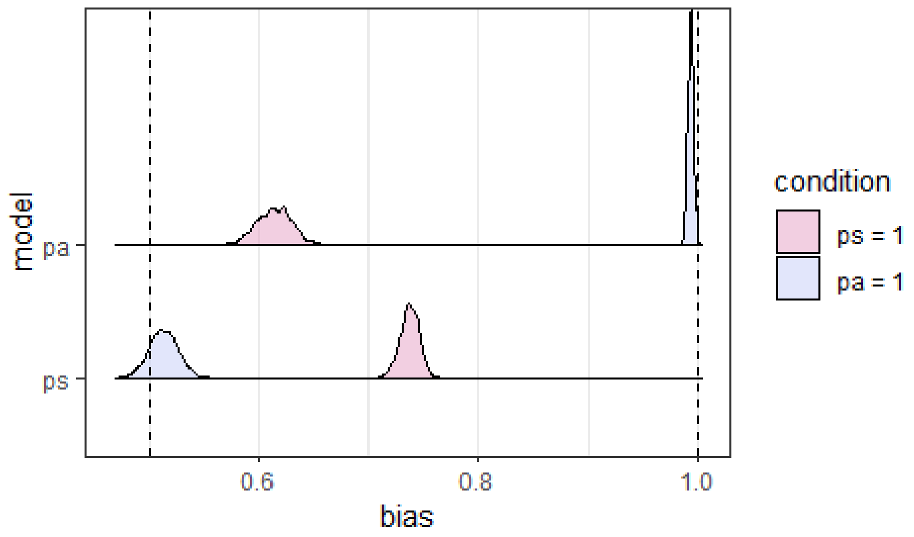

Fig 15: *Posterior plots for example with large p_s and large p_a biases, with a narrow prior*

Differentiation example: two conditions which give the same run statistics

In the following example, we set up a case with two conditions with different p_a and p_s values, yet that give the same run statistics. For example, in one run of the simulation, we can calculate the following ANOVAs:

```
## Anova Table (Type II tests)
##
## Response: n_runs
##              Sum Sq Df F value Pr(>F)
## fcondition        4  1    0.41   0.52
## Residuals       949 98

## Anova Table (Type II tests)
##
## Response: max_run_length
##              Sum Sq Df F value Pr(>F)
## fcondition        0  1    0.02    0.9
## Residuals       251 98
```

Table 8: *HDCI intervals.*

.width	ps_diff.lower	ps_diff.upper	pa_diff.lower	pa_diff.upper
0.53	-0.06	-0.04	0.12	0.15
0.97	-0.09	-0.02	0.10	0.17

Over 100 simulations, an ANOVA on the number of runs gives a statistically significant difference 8% of the time. An ANOVA on the maximum run length statistic gives a

statistically significant difference on 21% of the simulations. A significant difference in either statistic is detected only 29% of the time. In contract, our method finds a difference in p_a and p_s in 100% and 83% of simulations, respectively.

Misattribution example: a p_a bias with no p_s bias

A similar example can be seen when we have a p_a bias without a p_s bias. In this case, simple run length statistics will show a difference (see ANOVAs below), but it is not clear what cognitive process underlies them.

```
## Anova Table (Type II tests)
##
## Response: n_runs
##            Sum Sq Df F value  Pr(>F)
## fcondition   1156  1    91.1 1.2e-15 ***
## Residuals    1243 98
## ---
## Signif. codes:  0 '***' 0.001 '**' 0.01 '*' 0.05 '.' 0.1 ' ' 1

## Anova Table (Type II tests)
##
## Response: max_run_length
##            Sum Sq Df F value Pr(>F)
## fcondition    529  1    99.1 <2e-16 ***
## Residuals     523 98
## ---
## Signif. codes:  0 '***' 0.001 '**' 0.01 '*' 0.05 '.' 0.1 ' ' 1
```

Table 9: *HDCI intervals.*

.width	ps_diff.lower	ps_diff.upper	pa_diff.lower	pa_diff.upper
0.53	0.03	0.06	0.30	0.32
0.97	0.01	0.09	0.28	0.35

Over 100 simulations, our method finds a difference in p_a and p_s in 100% and 2% of simulations respectively, and thus is able to attribute the bias appropriately.

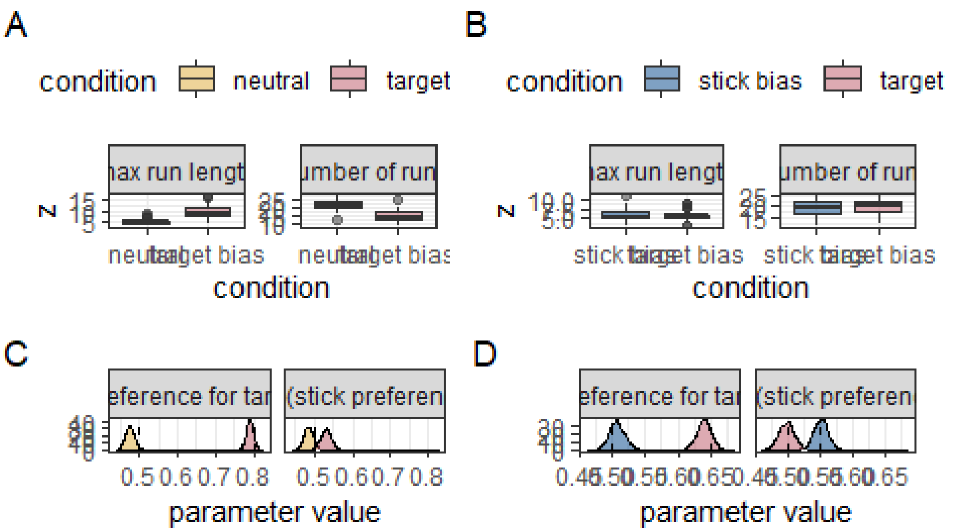

Fig 16: *Left top: boxplots showing the maximum run length and number of runs in each of our two simulated misattribution conditions. Left bottom: density plots showing the* p_a *and* p_s *values calculated by our model for each of the two simulated misattribution conditions. Right top: boxplots showing the maximum run length and number of runs in each of our two simulated differentiation conditions. Right bottom: density plots showing the* p_a *and* p_s *values calculated by our model for each of the two simulated differentiation conditions.*

Multi-level (multiple participants)

Simulation Example

First, we will show that we are able to recover overall p_s and p_a biases, as well as the parameters from individual participants.

Simulating Data

```
prob_a <- 0.67 # p(x=a | equal numbers of a and b balls left)
prob_s <- 0.75 # p(x_i = x_{i-1) | equal numbers of a and b balls left)

n_obs <- 20 # number of observers
n_trials <- 25 # number of trials per observer
n_targ <- 50 # the number of targets to find per trial
```

Summary stats

Here, we show the values of p_s and p_a for each observer, showing the individual differences between participants.

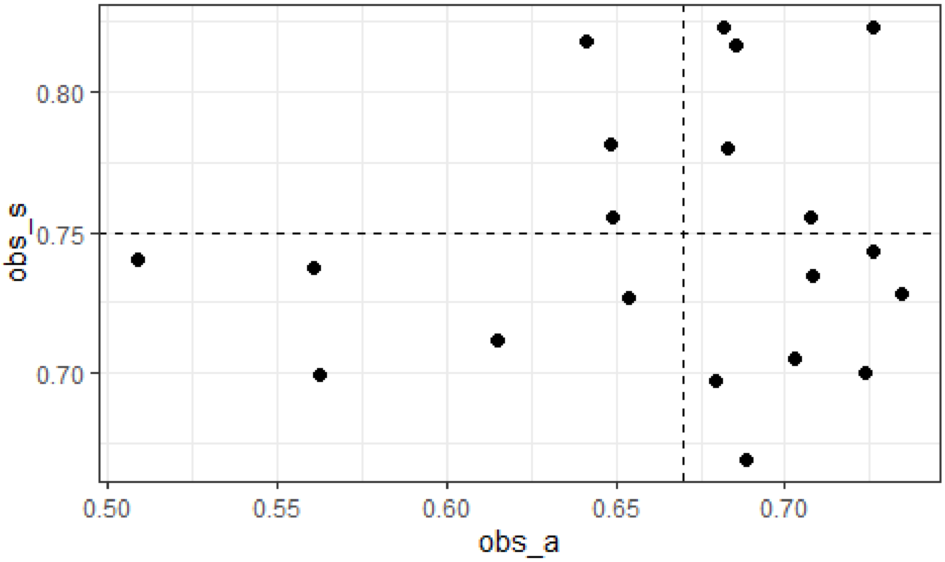

Fig 17: *Values of p_s and p_a for each individual in our simulated data.*

Model fitting

By fitting our multi-level model, we see we can recover our estimates of the overall p_s and p_a biases, as well as the estimates for each individual participant.

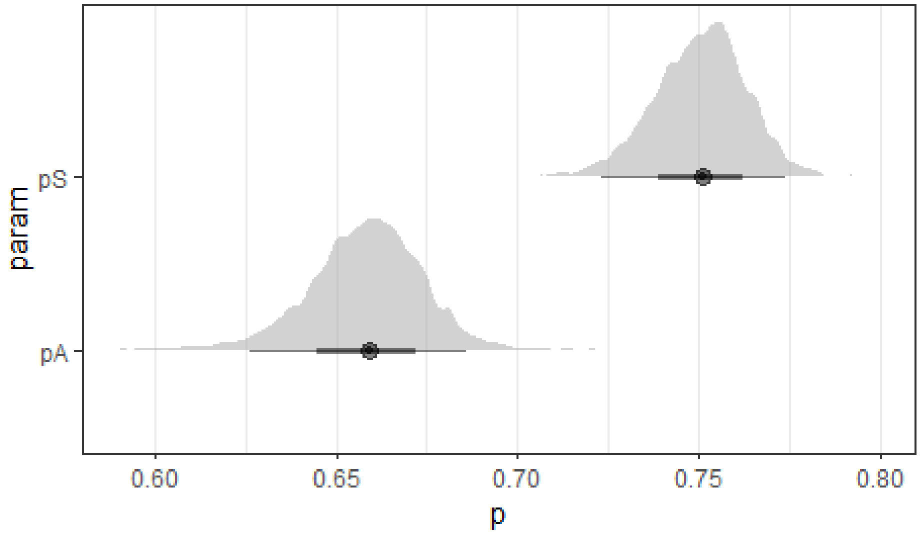

Fig 18: *Posterior plots for simulated data.*

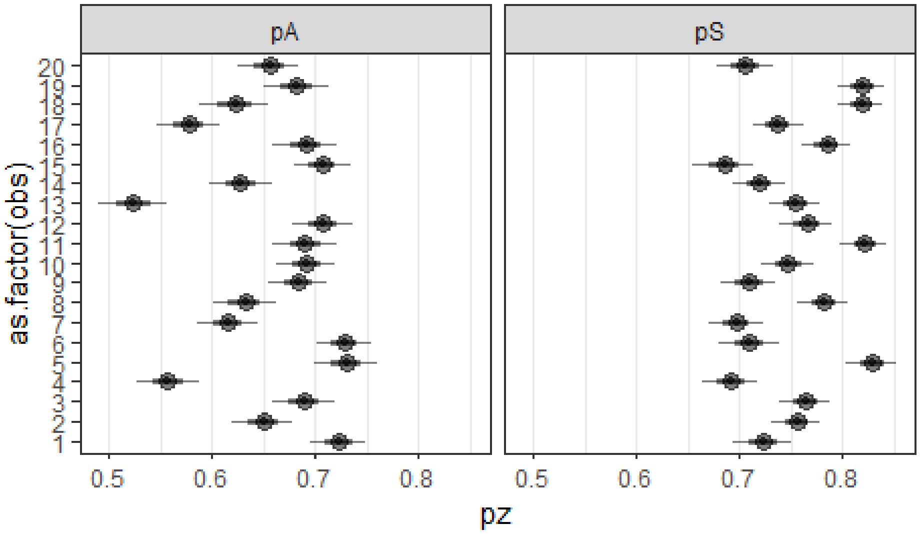

Fig 19: *Individual differences in simulated data.*

Real Data Examples

Kristjánsson et al (2014)

[2] was one of the first experiments to investigate multi-target search in humans, with an iPad based paradigm that involved participants 'foraging' for two different types of target (mixed in with two distractor types) by tapping on them sequentially. In the "feature" condition, the targets and distractors were discriminable using just colour as a cue (e.g. the targets were red and green, and the distractors were blue and yellow): in the "conjunction" condition, the categories were defined by both colour and shape (e.g. the targets were red squares and green triangles, and the distractors were red triangles and green squares). They found a now-classic pattern among their participants using the mean number of "runs" on a trial as their dependent variable: when search was easy, as in the feature condition, participants switched frequently between the two different target types. However, when search was more difficult, as in the conjunction condition, participants switched less frequently, leading to long "runs" of one target type.

Their experiment used 16 student participants, foraging for 40 targets on each of 20 trials. The dataset used for the following analysis was taken directly from the supplementary material of the journal article: we used trials 6-25 for each participant in each condition, as these were assumed to be the ones used in the original manuscript based on the description in the methods.

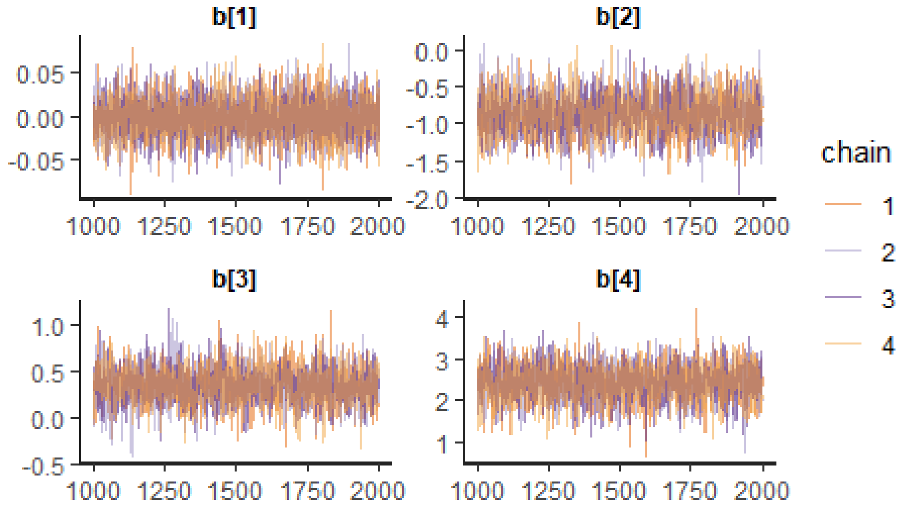

Fig 20: *Trace plots for re-analysis of [2]*

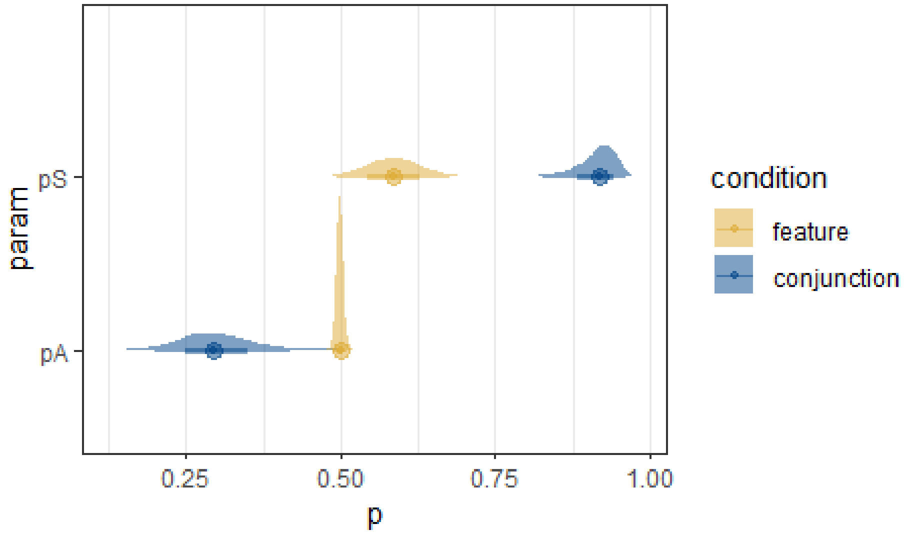

Fig 21: *Posterior plots for re-analysis of [2]*

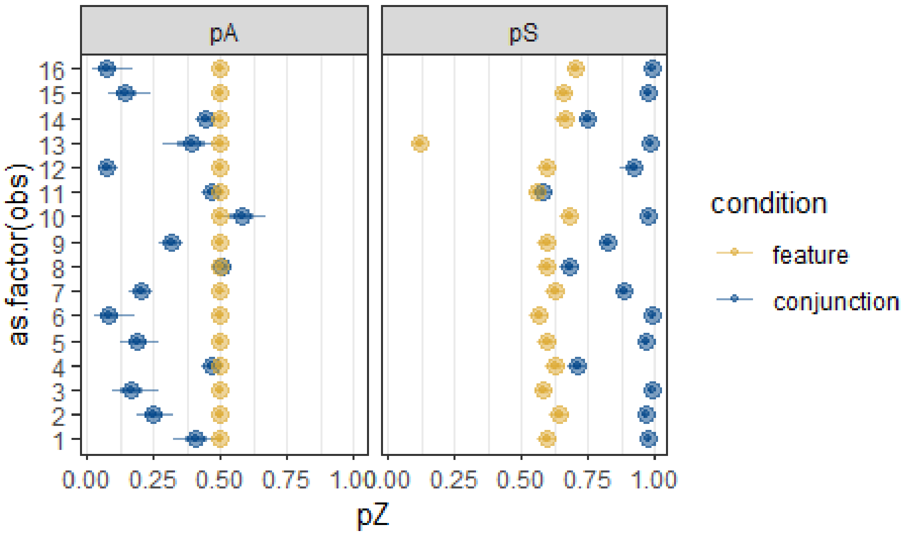

Fig 22: *Individual differences in re-analysis of [2]. Participants 4, 8, 11 and 14 are those identified as 'superforagers' in the original manuscript.*

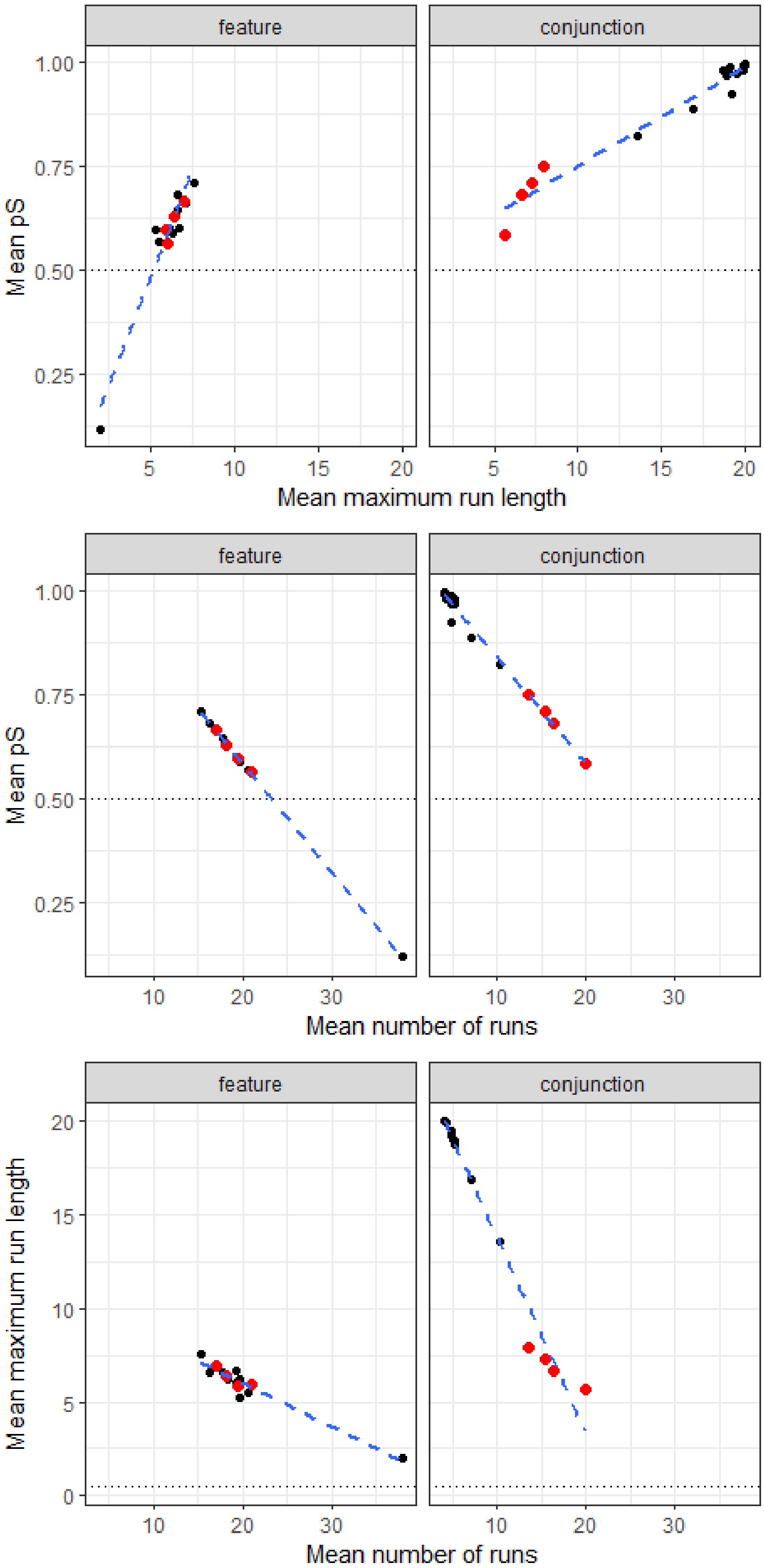
feature
conjunction
Mean pS
Mean maximum run length
Mean number of runs
Mean maximum run length
Mean number of runs

Fig 23: *Plots showing correlations of* p_s *with run length statistics in the re-analysis of [2]. Red dots show participants identified as 'superforagers' in the original manuscript.*

We can see that our p_s measure correlates well with the mean number of runs per participant, and the mean maximum run length.

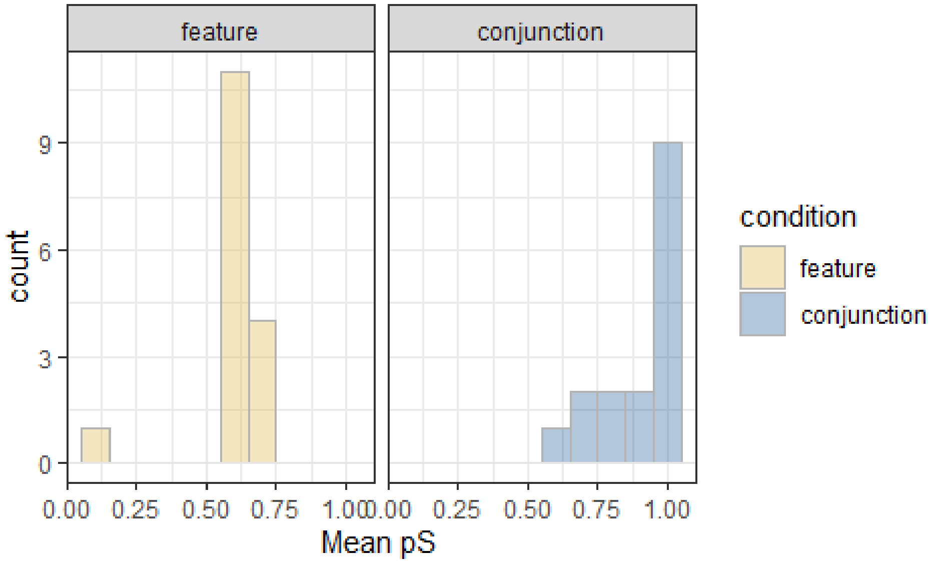

Fig 24: *Plot showing the distribution of mean* p_s *values across participants and conditions for re-analysis of [2].*

We can also see that p_s captures features identified in the original paper e.g. the presence of 'superforagers'.

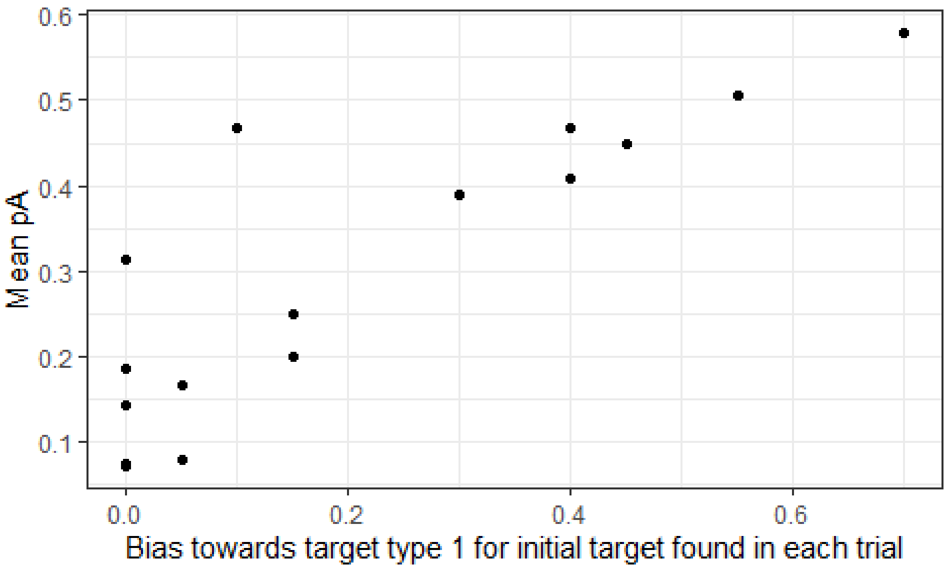

Fig 25: *Plot showing how mean* p_a *values vary with the proportion of times an individual selected target type 1 for the first target found in each trial in [2].*

Finally, p_a also seems to have a meaningful relationship with the stimuli: the higher the p_a, the more likely a participant is to select target type 1 as the initial target on each trial.

Tagu and Kristjánsson (2021)

[3] is a recent paper using a foraging paradigm, which we re-analysed because of their interesting manipulation involving value: in the 'value' blocks of the experiment, participants received more points for selecting a 'high value' target colour compared to 'low value' target colours. This condition thus manipulated participants' preference for a particular target type (compared to the 'no-value' block where all target types were equal). We would therefore predict that these two conditions would show different p_a biases in our framework.

We analysed only the mouse foraging blocks, and trials where the targets were randomly distributed (as opposed to in patches). Note that the paper had 3 different target types, but we have collapsed these into two target types for convenience. The dataset includes data from 24 participants, with 10 trials for each of the 'value' and 'no-value' blocks per participant, where they could find up to 54 targets on each trial (though they did not always have to find all the targets). For our reanalysis, we used 23 participants (we excluded one participant who did not complete all 10 trials).

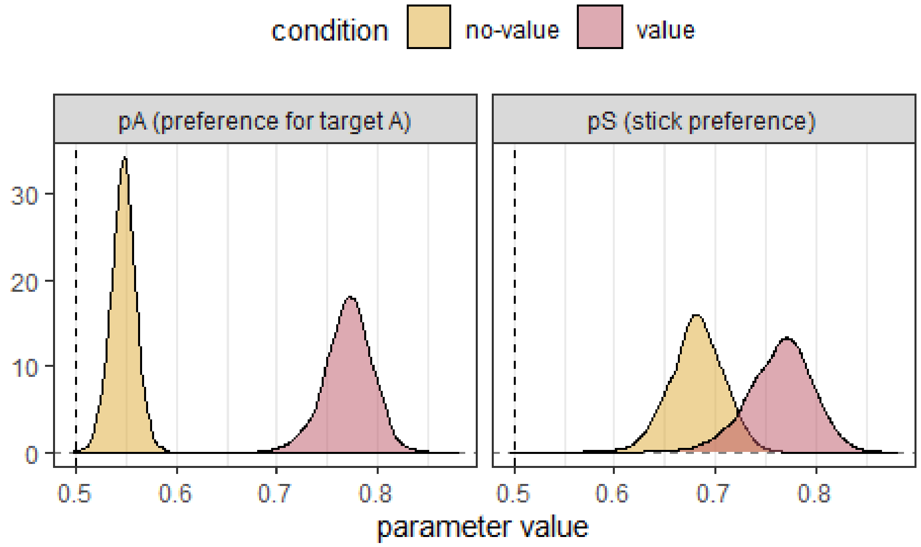

Fig 26: *p_a and p_s biases across participants in multi-level re-analysis of [3], showing both the value and no-value conditions.*

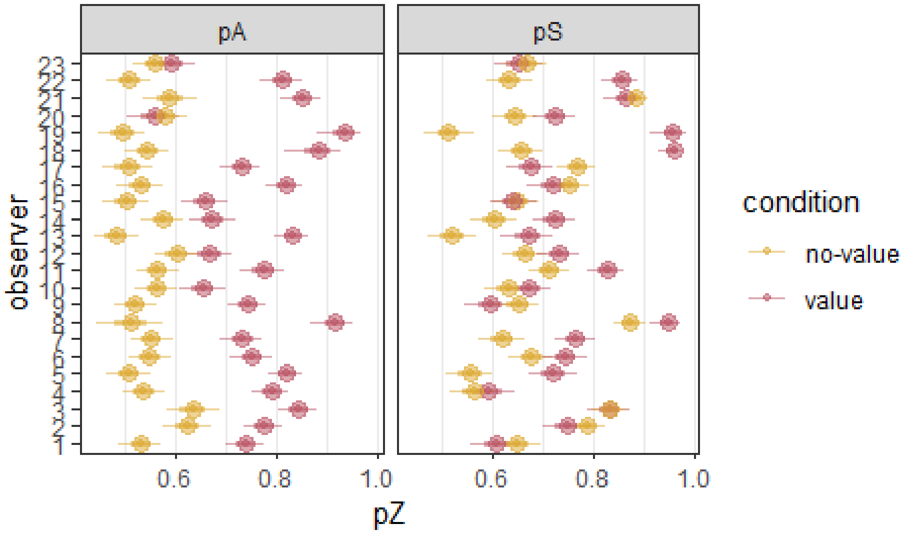

Fig 27: *p_a and p_s biases for individual participants in multi-level re-analysis of [3], showing both the value and no-value conditions.*

As expected, participants have higher p_a values in the value condition, though there is some variability in how sensitive participants are to the manipulation.

References

1. Dawkins M. Shifts of 'attention'in chicks during feeding. Animal Behaviour. 1971;19: 575–582.

2. Kristjánsson Á, Jóhannesson ÓI, Thornton IM. Common attentional constraints in visual foraging. PloS one. 2014;9: e100752.

3. Tagu J, Kristjánsson Á. The selection balance: Contrasting value, proximity and priming in a multitarget foraging task. PsyArXiv; 2021. doi:10.31234/osf.io/48pzy
