# Supplementary material for: Foraging as sampling without replacement: A Bayesian statistical model for estimating biases in target selection
Source: PLoS Comput Biol. 2022 Jan 24;18(1):e1009813. doi: 10.1371/journal.pcbi.1009813 (PMC8812991; doi:10.1371/journal.pcbi.1009813)
Supplement: S2 File — Supplementary materials for the spatial foraging model. (PDF) [file pcbi.1009813.s002.pdf]

Foraging for a Model: Part 2

A Clarke and A Hughes

05/03/2021

Spatial bias model

Simulated example of proximity bias

We first demonstrate a simple example of a proximity bias, using an example where both b_a and b_s are zero i.e. there are no target preferences or stick biases. However, the simulated participant does have a preference for selecting a target near to the previous one found.

```
n_trials = 50
n_targets = 40
n_found = 40

tune_prox = 15
tune_angle = 0
bA = 0
bS = 0
```

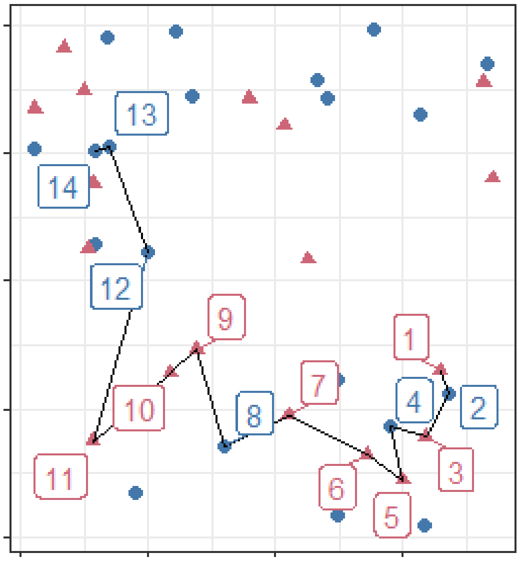

***Fig 1**: An example trial from our simulated data.*

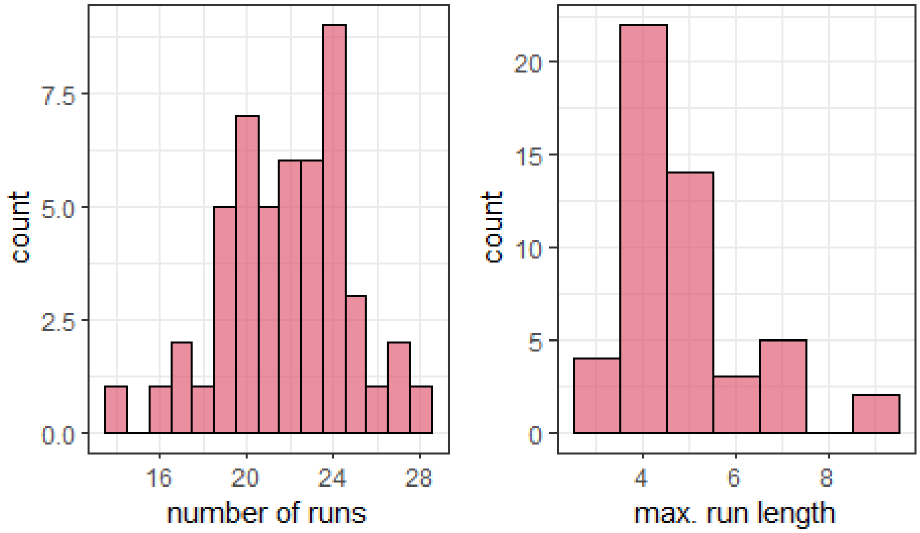

Fig 2: *Left: number of runs in simulated data. Right: max run length in simulated data.*

Our simulated data has the following statistics:

Mean number of runs = 21.82

Mean max run length = 4.82

Upon fitting our model, we can see that it returns good parameter estimates.

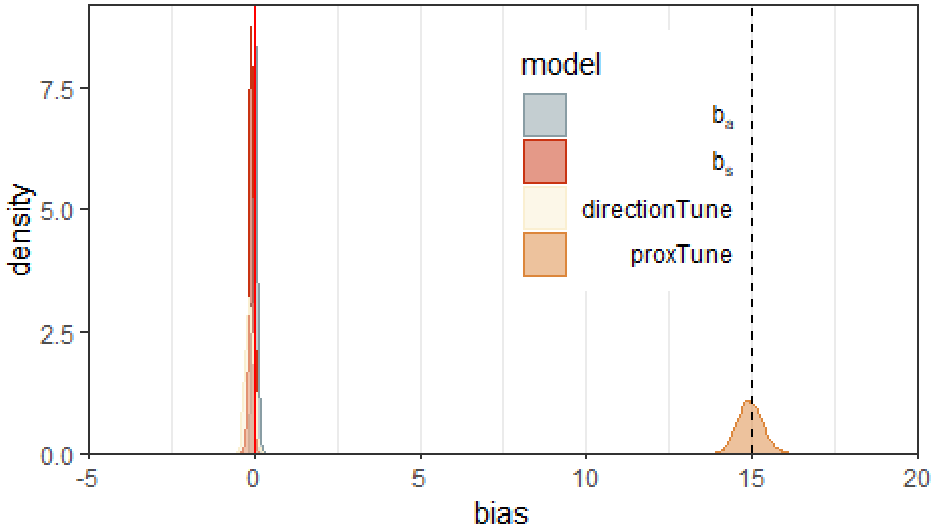

Fig 3: *Posterior plots for simulated proximity bias example.*

Clumped target example

One interesting case is where the targets are highly clumped, as might be found in natural environments (e.g. berries found on a bush). When using run statistics, it may appear that the participants have a stick bias, when they in fact have only a preference for selecting the nearest target.

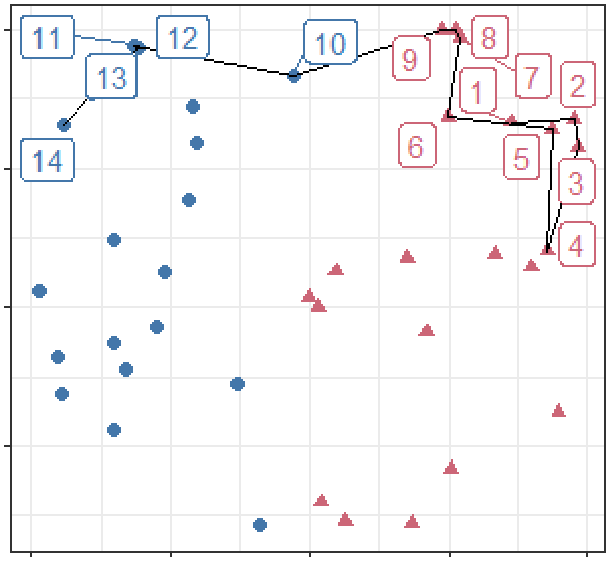

Fig 4: *An example trial for a clumped target display.*

From the run length statistics, we can see that this example has fewer and longer runs compared to the simple example above without clumped targets:

Mean number of runs = 6.68

Mean max run length = 14.24

Modelling with and without proximity bias - comparing the spatial and bag model

If we use a simple bag model, without incorporating a proximity bias, we can see that our model incorrectly predicts a p_s bias. However, with a spatial model, it is correctly able to recover unbiased p_a and p_s values, as well as a strong proximity bias.

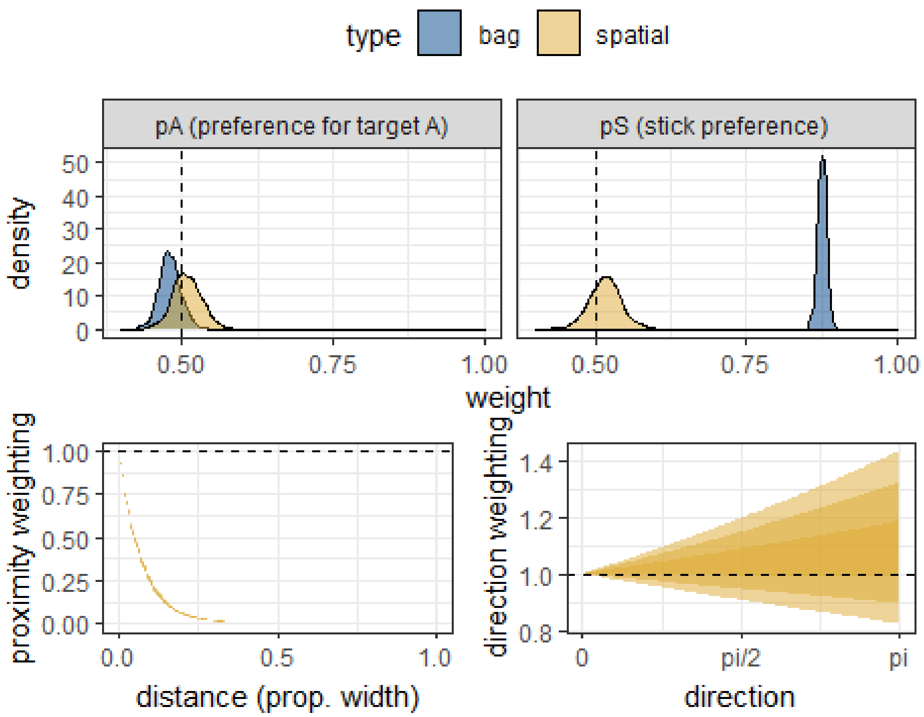

Fig 5: *Posterior distributions for both a bag model (without proximity bias) and a spatial model (with proximity bias) trained on patchy stimuli, where target types are clumped.*

Adding in a direction bias

We can also model a direction bias. A positive bias means that people are more likely to select the next target carrying on in the same direction; a negative bias means that they are more likely to double back on themselves, creating 'spiky' patterns.

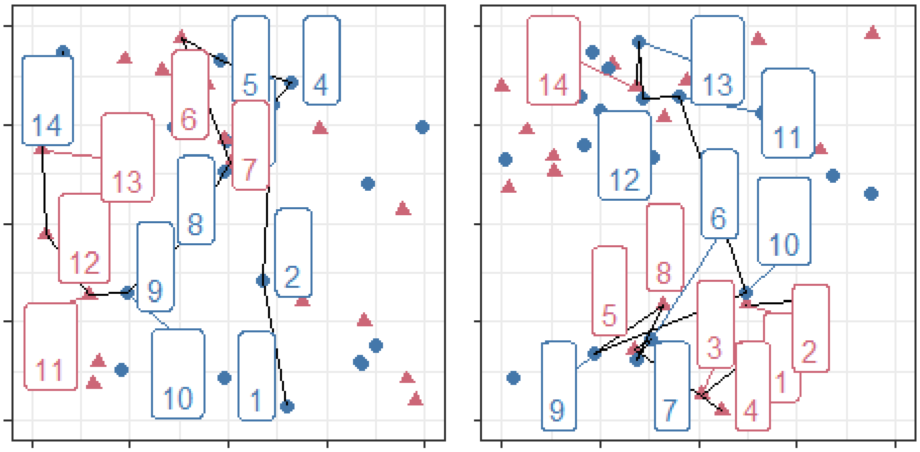

Fig 6: *Left: simulated example of a positive direction bias. Right: simulated example of a negative direction bias.*

Tune_angle = 5

As before, our model can easily recover the correct parameters (parameter b[4] is the direction bias):

```
## Inference for Stan model: momentum_foraging.
## 1 chains, each with iter=2000; warmup=1000; thin=1;
## post-warmup draws per chain=1000, total post-warmup draws=1000.
##
##                   mean se_mean     sd    2.5%     25%     50%     75%
98%
## b[1]              0.01    0.00   0.06   -0.11   -0.03    0.01    0.05
0.13
## b[2]              0.02    0.00   0.06   -0.10   -0.02    0.02    0.06
0.15
## b[3]             14.61    0.01   0.36   13.89   14.36   14.61   14.88
15.29
## b[4]              4.89    0.01   0.18    4.56    4.78    4.89    5.01
5.25
## prox_prior       88.70    3.94 113.25    7.16   28.68   56.52  110.91    3
30.82
## lp__          -2379.06    0.06   1.49 -2382.60 -2379.80 -2378.74 -2377.99 -23
77.20
##              n_eff Rhat
## b[1]          1025    1
## b[2]           937    1
## b[3]           860    1
## b[4]           866    1
## prox_prior     827    1
```

```
## lp__          578    1
##
## Samples were drawn using NUTS(diag_e) at Tue Jan 18 10:42:16 2022.
## For each parameter, n_eff is a crude measure of effective sample size,
## and Rhat is the potential scale reduction factor on split chains (at
## convergence, Rhat=1).
```

Tune_angle = -5

And again, our model can easily recover the correct parameters (again, b[4] is the direction bias):

```
## Inference for Stan model: momentum_foraging.
## 1 chains, each with iter=2000; warmup=1000; thin=1;
## post-warmup draws per chain=1000, total post-warmup draws=1000.
##
##                 mean se_mean    sd     2.5%      25%      50%      75%     
98%
## b[1]            0.09    0.00  0.06    -0.02     0.06     0.09     0.13     
0.21
## b[2]            0.09    0.00  0.06    -0.03     0.05     0.09     0.14     
0.21
## b[3]           14.28    0.01  0.32    13.68    14.06    14.27    14.49    1
4.90
## b[4]           -4.88    0.01  0.15    -5.18    -4.98    -4.87    -4.77    -
4.60
## prox_prior     82.99    3.12 94.69     7.62    27.06    51.73   103.82   39
6.39
## lp__        -2516.66    0.05  1.30 -2519.83 -2517.29 -2516.38 -2515.73 -251
5.00
##             n_eff Rhat
## b[1]          841    1
## b[2]          831    1
## b[3]          797    1
## b[4]          910    1
## prox_prior    924    1
## lp__          609    1
##
## Samples were drawn using NUTS(diag_e) at Tue Jan 18 10:48:34 2022.
## For each parameter, n_eff is a crude measure of effective sample size,
## and Rhat is the potential scale reduction factor on split chains (at
## convergence, Rhat=1).
```

Multi-level spatial models

Here, we show a number of examples using our full, multi-level spatial models on real datasets.

Kristjánsson et al (2014)

First, we re-run the [1] data we ran with the bag model, updating to the full spatial model.

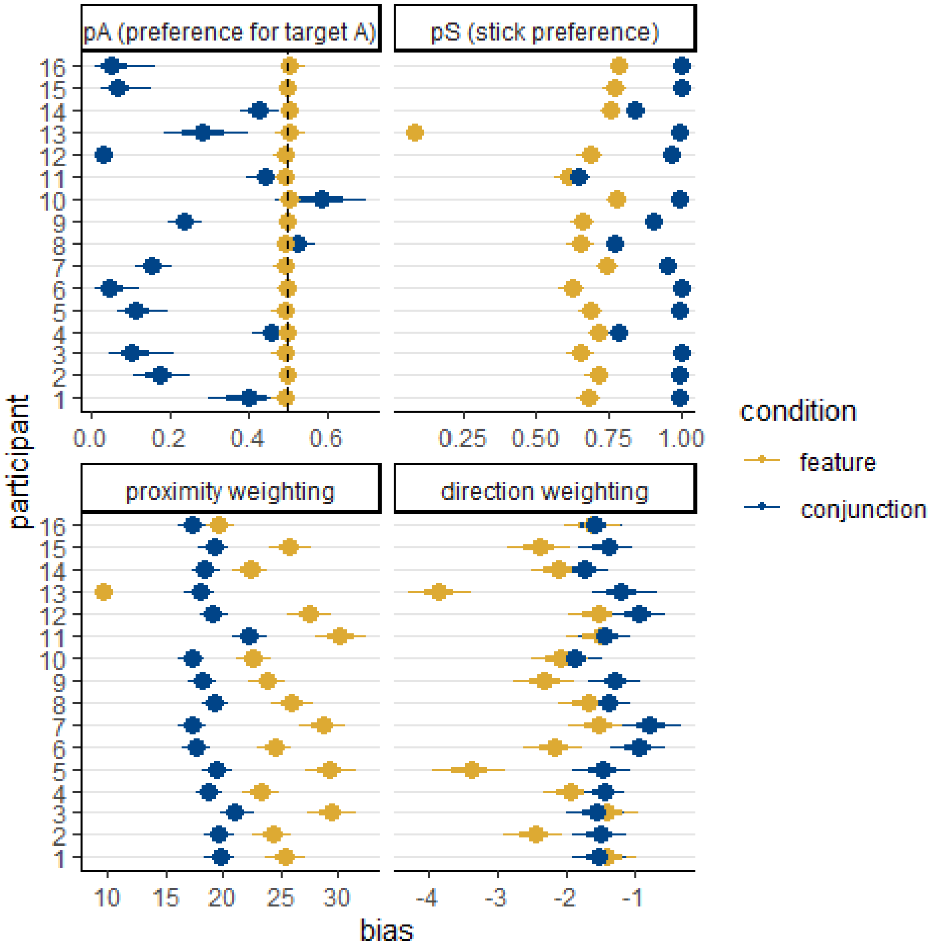

Fig 7: *Spatial model fit to [1], showing individual differences in parameter values.*

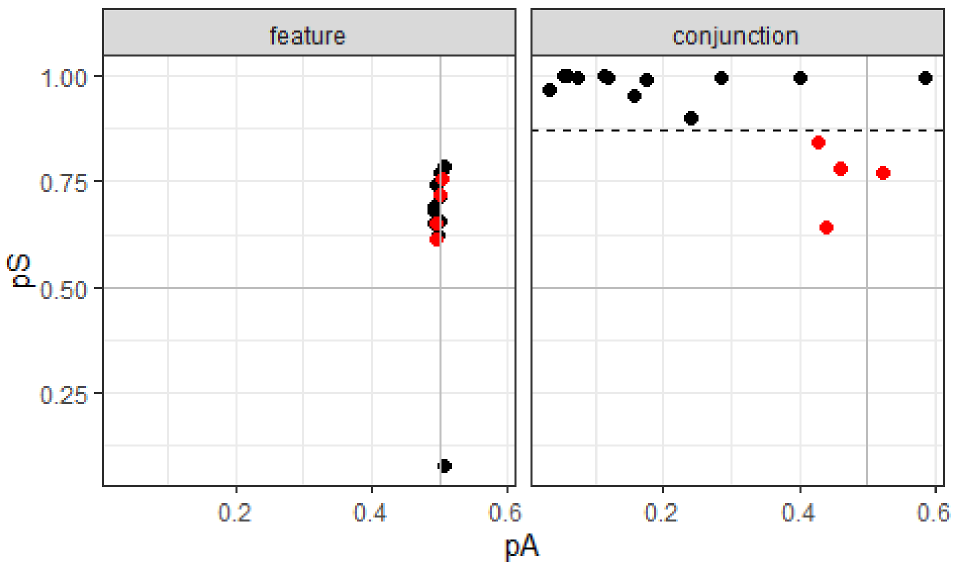

Fig 8: *Correlations between p_s and p_a in [1] model fit. 'Super-foragers' are marked in red.*

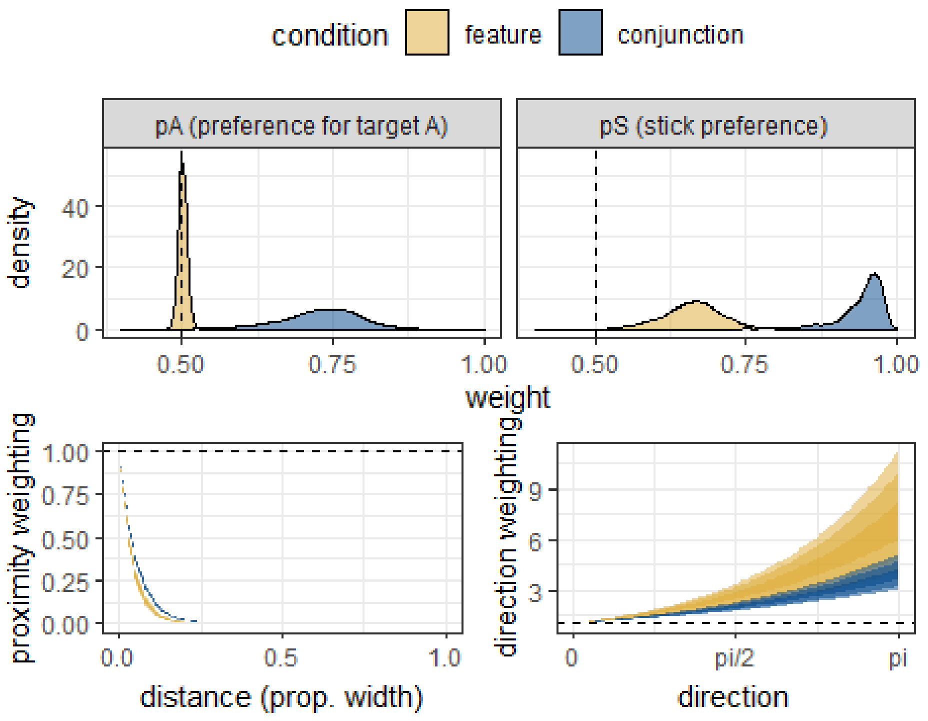

Fig 9: *Posterior distributions for our model when trained on data from [1].*

Kristjánsson et al (2014) - one trial

One of the strengths of our model is that it is able to recover good parameter estimates, even with a relatively small dataset. In the following fit, we use just the first trial per

condition per participant from the Kristjánsson et al (2014) data, and are able to obtain very similar estimates to the full dataset.

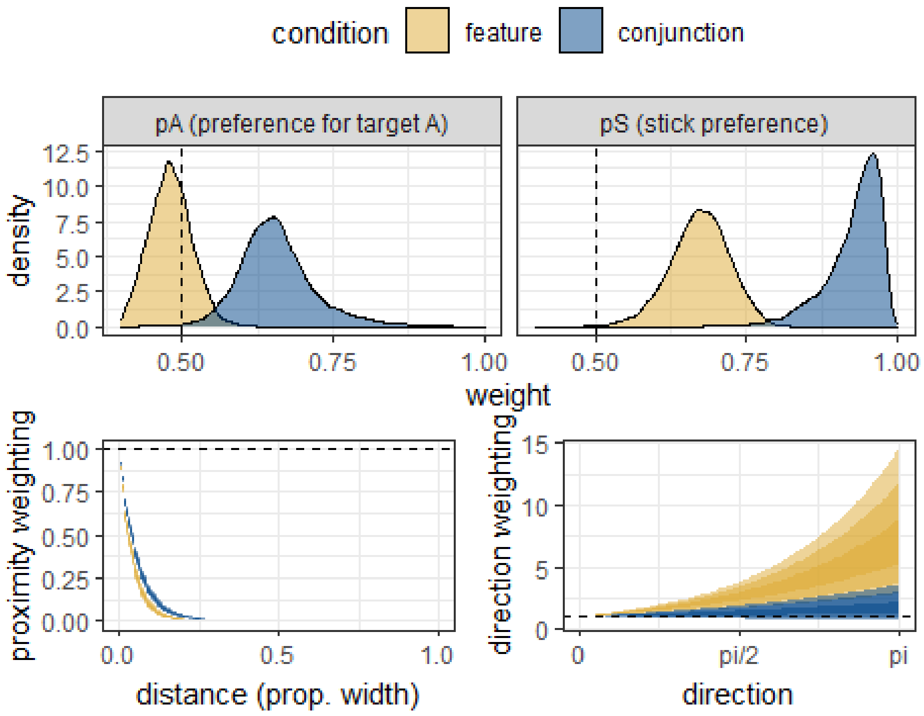

Fig 10: *Posterior distributions for our model when trained on data from [1], with just one trial per condition per person.*

Clarke et al (2018)

The [2] dataset contains a near-replicate of the [1] study, except using a larger number of participants: we used 58 participants in our re-analysis (the same number as in the original paper, except that we also removed one additional participant that was labelled as being a different version of the experiment).

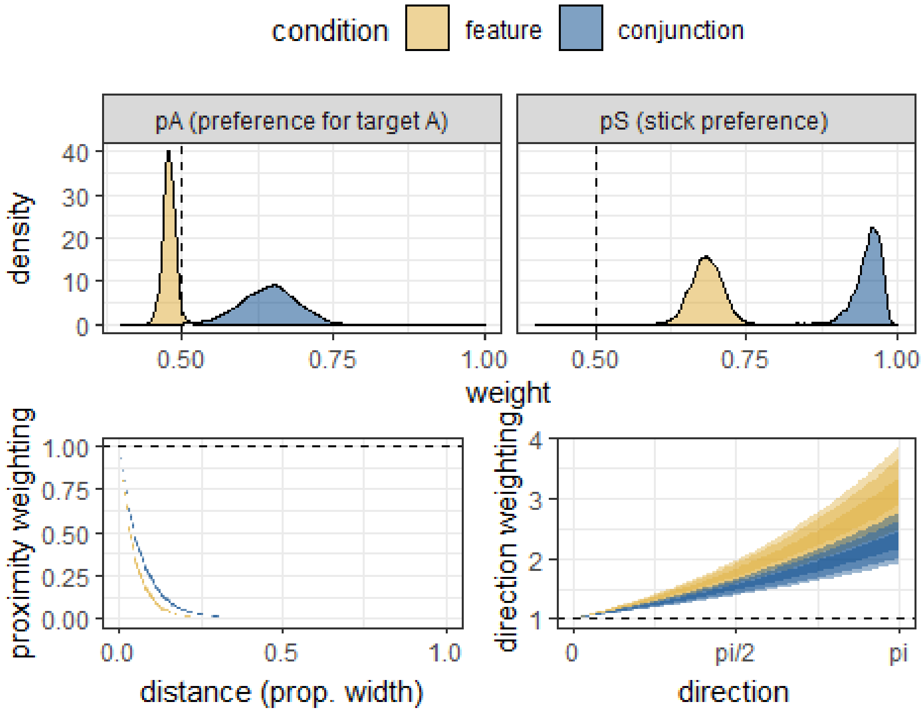

Fig 11: *Posterior distributions for our model when trained on data from [2].*

Thornton et al (2020)

Finally, we consider a recent dataset from Thornton and colleagues, where they investigate "foraging tempo", the rate of successive target selections, using the same target types as used in [1], with 11 participants. While our model at present does not directly include this parameter and is not able to take into account the staircase procedure they used, we split the dataset into "slow" and "fast" foraging tempos (by taking the 5 trials with the lowest foraging tempo and the 5 trials with the highest foraging tempo, respectively) and treated these as two discrete conditions. We can see quite clearly from our model plot that the "fast" condition tends to lead to participants sticking with the same target type more, while not adjusting any of the other biases.

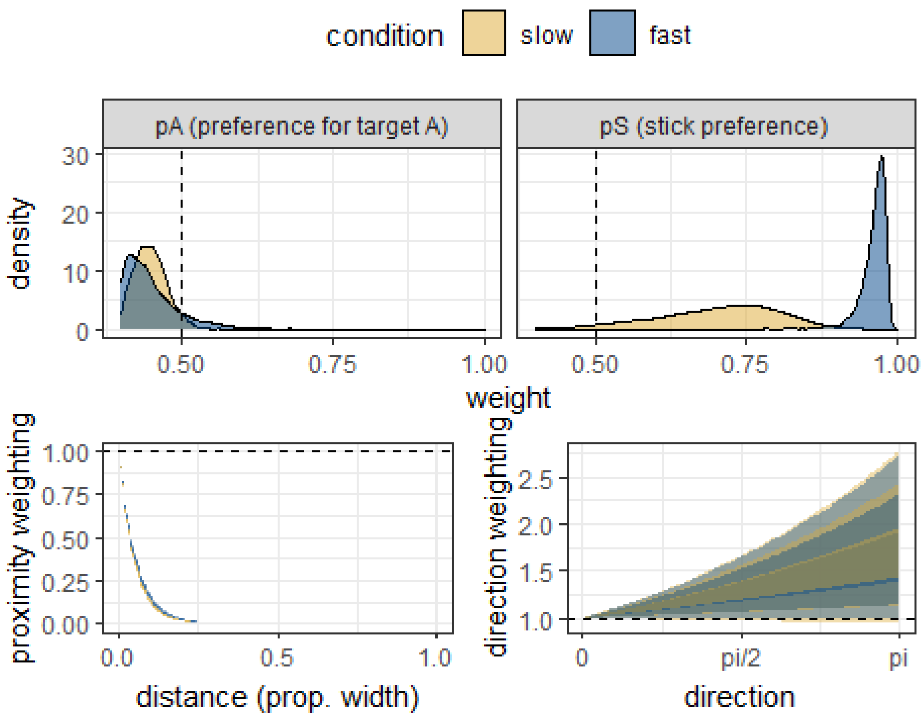

Fig 12: *Posterior distributions for our model when trained on data from [3].*

References

1. Kristjánsson Á, Jóhannesson ÓI, Thornton IM. Common attentional constraints in visual foraging. PloS one. 2014;9: e100752.

2. Clarke AD, Irons JL, James W, Leber AB, Hunt AR. Stable individual differences in strategies within, but not between, visual search tasks. Quarterly Journal of Experimental Psychology. 2018; 1747021820929190.

3. Thornton IM, Nguyen TT, Kristjánsson Á. Foraging tempo: Human run patterns in multiple-target search are constrained by the rate of successive responses. Quarterly Journal of Experimental Psychology. 2020; 1747021820961640.
