# Supplementary material for: Foraging as sampling without replacement: A Bayesian statistical model for estimating biases in target selection
Source: PLoS Comput Biol. 2022 Jan 24;18(1):e1009813. doi: 10.1371/journal.pcbi.1009813 (PMC8812991; doi:10.1371/journal.pcbi.1009813)
Supplement: Attachment — Submitted filename: foraging_responsetoreviewers.docx [file pcbi.1009813.s008.docx]

***We thank all the reviewers for their extremely helpful and constructive comments. Our responses are in bold italics below.***

Reviewer #1: The paper Building Bayesian Cognitive Models of Visual Foraging by Clarke et al. introduces a new approach to modeling data from visual foraging tasks. Importantly, the approach can disentangle different influences on target selections and predict e.g. run-behavior in foraging for different conditions. The model is a much-needed tool for a field that lacks adequate methods to deal with the rich data produced by foraging tasks.

The paper is well structured, the rationale is clear, and the model and its applications are presented in a comprehensible manner. I only have a few minor points and suggestions, which I list below (not necessarily in order of importance).

* p2: "... below the average rate of return across all patches". - I suggest adding something to highlight that the avg. ROR across all patches also includes travel time during which no elements can be collected. Sometimes people not familiar with MVT are confused about this ...

***Thank you for suggesting this clarification, we have incorporated it on line 16.***

* p2: The idea of a "search image" is used on p2. Perhaps after this passage, you could mention the related concepts of attentional templates concerning visual search in humans. The concept of templates appears later in the current manuscript but was nowhere introduced ... this might be a possibility.

***This is a good point, and we have now incorporated a definition of ‘attentional template’ into this paragraph (lines 29-31).***

* On p2 you suggest that foragers can keep "multiple target templates active in working memory". On p3 you write " potentially implying that they are able to hold multiple target templates simultaneously in working memory", as a special case for super-foragers, implying that normally this might not be possible (somewhat in conflict with what was said on p2). I think the topic is still debated. The point here could probably be made without committing to either view.

***We have reworded the text on P2 (lines 33-34) to indicate that it’s the switching between target templates which is the cost (and then it is hopefully clearer later that for superforagers, this switching may not be necessary, leading to more efficient searches).***

~ p4: It does not become clear that (and why) the model focuses on a binary case with two target types. Also, in the discussion, where limitations are discussed, this point is not taken up either.

***We have indicated more explicitly that our model is binary on P4 (line 108), and have now mentioned this as something that could be extended in further versions of the model in the discussion (lines 355-358). At the time of writing our paper, the majority of studies and available datasets concentrated on only two target types, and as such, this was the focus of our manuscript. However, it would be conceptually trivial (although a little bit fiddly in practice!) to update the model so that it can take n>2 target types into account. We opted to leave such details to a future paper.***

* On several occasions, you point out that existing methods might not adequately model the underlying cognitive processes, implying that your approach does. At one location, it is also explicit: "A strength of our model is it allows us to more accurately determine the cognitive processes underlying foraging patterns." While I completely agree that your model is a significant step forward compared to earlier methods and goes a long way in disentangling different biases, I would say that it still does not "determine the cognitive processes underlying foraging patterns." How do these processes lead to a particular switching frequency? Why is there a preference for certain features? ....

***We have softened our wording in the example given (lines 159-160). We agree that our model cannot help us understand the neural basis of these processes (the ‘how’ questions), but arguably by incorporating all the relevant parameters (and we agree that our model probably doesn’t yet!) the model in theory should be able to provide a good account of the cognitive processes involved in foraging when extended: we make the point in the conclusion that the pA bias could be broken down into sub-biases for colour and  luminance, thus helping to answer why there is a preference for certain features which could be related to other modelling frameworks (e.g. salience models).***

* p8: Suddenly, the term "bag model" appears. It also occurs as "bag foraging model", sometimes set in italics. Perhaps this term should be introduced earlier.

***Thank you for picking up on this, we have now standardised this to ‘bag foraging model’ (first introduced on line 107).***

* p5: Figure 3 refers to "salience" (and it comes up later again). The main text does not introduce the idea that salience could drive a target tepy preference. The real-data example uses the value as the reason for preferences …

***Thank you for pointing this out, we have now renamed this to a ‘target bias’ and elaborated on what we mean by this in the main text (lines 161-163).***

* p5: Figure 2: "the chicken data" sounds a bit odd. Perhaps add a reference?

***This has now been corrected.***

* p7: I find the term "conflated power example" unclear.

***We have reworded this to ‘differentiation example’, which is hopefully clearer (line 171).***

* p9: "identical to the training data": I think the ML framing (which only appears here) could be confusing for some readers

***We have reworded this to try to make this clearer for all readers (lines 225-227).***

* p11: "leading the model to favor doubling back on itself to select targets.". I have only a vague idea of what this means ... perhaps a figure could be helpful here.

***These figures are available in the supplementary material: we have referenced them more directly here (lines 283-284).***

* p11: what is an "eligible target". Aren't all targets eligible?

***We have removed eligible here (line 290) - though in the standard human foraging studies, there are distractor targets that are in effect ineligible because clicking on them ends the trial. However, as our model currently doesn’t incorporate any notion of distractors, we agree it is not helpful to refer to eligible in this context.***

* p15 (an also the title): "Bayesian cognitive model" - I know this term is used for different things (and e.g. in the Lee and Wagenmakers book in a similar context as here). Personally, I try to avoid it because people easily confuse it with Bayesian models of cognition, i.e. the idea that cognitive processes follow Bayesian principles. If I see it correctly, your study does not suggest this (of course, it might also not exclude it) but simply uses a Bayesian statistical framework.

***We have modified the title to ‘Foraging as sampling without replacement: a Bayesian statistical model for estimating biases in target selection’.***

* The paper contains many typos. Here are some I spotted:

***Apologies, these have now been fixed (see marked up document).***

- p1, 1st para: missing closing parenthesis

- Figure 1: Caption says "top" and "bottom," but panels are on the left and right.

- Figure 1: I think "first three trials" should be "first three selections"

- p5: HDPIs --> HPDIs, also the acronym is never introduced; in Figure 5 you use "HDI".

- p5: Footnote: consider removing point iv). People not very familiar with Bayesian stats might think this might really be of relevance

- p6: "(5%" - closing parenthesis missing

- p6: "our model__s__ then becomes"

- p7: "seen in 3" ... "Figure" is missing

- "super-foragers" vs. "superforagers"

- P10: "Figure Figure 5"

- p11: "to appear to be"

Reviewer #2: I was impressed with this manuscript. It is written very clearly and efficiently and presents to me what seems like a very useful approach to understanding foraging. While I do think that accounts based on run behavior can still definitely aid our understanding of foraging in particular and visual attention and attentional selection more generally, the current approach has considerable merits, which the authors make a very clear and concise case for.

I therefore do not have many comments, and my comments therefore can be taken as food for thought.

Firstly, I would recommend not talking about "confounds" with regard to the literature that has focused mostly on selection runs. Yes, it is true that run length, run number, switch rates are interdependent and correlated, but I do not see the problem that the word confound implies. As I see this, the current authors sampling approach can well hold it’s own without trying to imply that run based approaches need to be avoided as language such as confounds certainly implies. The uncertainty with regard to cognitive mechanisms behind run behavior is something that the authors could focus more in in my opinion – for example the finding that children under 12 tend not to switch, may point towards mechanisms.

***We have removed the phrasing of ‘confounding’ as suggested (see abstract). We agree that the cognitive mechanisms behind run behaviour are very interesting, and have tried to highlight this more in the introduction, in reference to the findings that children under 12 tend not to switch (lines 61-64).***

When the authors talk about how optimal foraging theory has sometimes run into problems, I thought they should mention a recent finding by Kristjánsson, Björnsson & Kristjánsson ([https://doi.org/10.3758/s13414-019-01941-y](https://linkprotect.cudasvc.com/url?a=https%3a%2f%2fdoi.org%2f10.3758%2fs13414-019-01941-y&c=E,1,-HeoKh25FOMg2NOWqVflpKdXm1vuQZFjArXx2ex1TdWh8SWKS9QBMVIcIoUr3s5al9hBflyaIcbVPozZPkZxm7By0d0eJoqBnDt_NmoU8NHFhbaQEBT9ce4HTg,,&typo=1&ancr_add=1) ) where the authors claim that human foraging in paradigms in many ways identical as those under investigation in the manuscript here deviated from the predictions of optimal foraging conceptions and that participants kept on foraging within the same patch much longer than expected. The authors concluded that human foraging is probably influenced by too many factors to be captured with a relatively simple mathematical model. This actually speaks to what my main point about this paper is in that there are so many factors at play that determine foraging performance that capturing them all within a single model is probably premature, if not impossible.

***Thank you for pointing us towards this very interesting paper. We have cited it in the introduction, in reference to MVT (lines 18-19), and also in the discussion, where we recognise that a ‘unified theory of foraging’ is likely to be a challenging task! (Lines 365-367).***

With regard to this issue of „patch leaving“, I encoourage the authors to provide a little more detail and be a bit more explicit in the discussion about how this could be implemented within their model. This is only briefly metnioned in the current discussion.

***If we take a trial as a “patch”, then a stopping rule is also a patch leaving rule. A simple stopping rule could be implemented in many different ways. For example, after each target selection, there could be some chance to terminate the search (this probability could be constant, or could increase with the number of targets already found in the trial). Fitting such parameters to empirical data would be an interesting development of our model.***

***Alternatively, if we are thinking about having patches made up of multiple “clumped” targets on a single large display, we could modify the spatial bias component of our model to include a categorical factors indicating the different clumps of targets (i.e. targets in different patches are in some sense perceptually further away from each other).***

***We have added more to the discussion about these ideas (lines 345-352).***

I went through the mathematical modelling and it seemed to be very solid.

I do share the authors enthusiasm with regard to cluttered scenes. It would for example be highly interesting to vary noise levels – and in fact many means are possible it seems.

Overall, I commend the authors on what is in my opinion a very fine contribution to the literature.

***Thank you very much for your kind comments.***

Reviewer #4: This paper describes two versions of a model of visual foraging aiming at providing more details about foraging behaviour than the run statistics often used in the literature. Notably, run statistics can inform about the tendency to stick with one target type or to switch, while the “pulling balls out of a bag” model offers the possibility to assess pA (the preference for one target type or another) on the top of pS (the tendency to stick with one target type or to switch). The second version of the model allows assessment of spatial biases with two additional parameters for proximity and direction.

I enjoyed reading this paper. It is nicely written, and the paper is well motivated and of high interest in the field of visual foraging. The maths is explained in a very pedagogical way so that it is also understandable by non-experts. Moreover, I wish to thank the authors for providing useful, detailed and well-constructed supplementary materials on the top of the main article.

I only have a few comments below that I hope are useful.

Jerome Tagu

1. I find the four parameters described in the paper useful for assessing foraging behaviour, but I feel they are presented independently, in separate analyses (especially the spatial biases that seem separate from the selection biases). I wonder whether it would be possible to have the four parameters in a single analysis, or equation (a bit as we would have in a regression analysis), so that the behaviour of a given individual could be summarised in a very straightforward way (e.g. this participant’s behaviour is explained at 80% by a preference for target A and at 20% by a preference for proximity). NB: I’m not an expert in Bayesian modelling, so maybe this is something not doable, but if doable, it would be very cool.

***Apologies if this was not clear in the manuscript: the spatial model builds upon the bag model, so it contains pS and pA as well as the new spatial parameters (e.g. as can be seen in Figure 5). We have reworded the introduction to the spatial models section to hopefully make this clearer (line 245).***

2. It is said multiple times (l.84-87, l.306-311, and probably elsewhere), that the model can generate good parameter estimates with little trials. Is the model also resistant to the manipulation of the number of target selections per trial? Knowing this would be very useful for the study of populations such as children, who have difficulties to perform many target selections per trial (see, e.g. Olafsdottir et al., 2016, 2020).

***This is something we look at in the supplementary material (part 1, section 1.4) in relation to the chicken foraging data (Dawkins, 1971).  We found that the number of target selections didn’t have a huge impact on the estimation of pS. As you say, an alternative option is to have a relatively high number of target selections, but only one trial: in the supplementary material (part 2, section 1.4.2) we show that we are able to obtain pretty good parameter estimates for Kristjánsson et al (2014) using just one trial of data per participant. So we think our modelling may be a good option for experiments where it is difficult to collect a lot of data from participants (as we discuss on P13, L320-322). Our model is also generative, and thus can easily be used for simulation based power analysis for new experiments to check the impact of e.g. number of trials, or number of target selections on the specific study being proposed.***

3. I understand that the model is somehow limited by the number of target types (can only be used with two target types). More and more studies are now contrasting 3+ target types, and I would find useful to update the model so that it could be used to assess the relative preference (pA) for n = 3 or more target types.

***We agree, and this is something we hope to be able to do in future versions of the model. We would love to collaborate if you have suitable datasets to suggest! In the current manuscript, we have added a section to the discussion to make it clear that for now, the model only works with two target categories (lines 355-358).***

4. In the re-analysis of the data from Kristjansson et al. (2014), the model allows a clear identification of “super-foragers” who show low pS in conjunction foraging (e.g. Fig.21 of the supplementary materials part 1). Just to be sure: are these super foragers the same individuals as the ones identified as super foragers in the original analysis? Similarly, are the participants showing a strong preference for valuable targets on supplementary figure 26 the same individuals as the ones identified in the original analysis in Tagu & Kristjansson (2022)?

***Yes, these are indeed the same individuals who were highlighted as superforagers in the original paper. The supplementary material has now been updated to make this clear. We would love to compare our individual-level  findings with Tagu & Kristjansson (2022) as well, but as far as we can tell, the participant level data in Figure 3 has been re-ordered and we are not sure how this corresponds to the participant labelling in the original data file. If you would be willing to share this conversion with us (or point us in the right direction if we have missed the information - apologies if so!) we would be extremely happy to check further.***

5. Data used for testing the model were imported from studies involving “exhaustive” foraging (e.g. Kristjansson et al., 2014), where participants were required to select all available targets, as well as from studies involving “non-exhaustive” foraging (Dawkins, 1971; Tagu & Kristjansson, 2022), where participants only select some of the available targets. If I understand correctly, the model doesn't seem to be influenced by the differences in task instructions and can reliably generate pS and pA in both conditions. Maybe the authors would like to highlight this other strength of the model, on the top of its other qualities.

***We discuss non-exhaustive foraging briefly on lines 345-348 - we think that to really model this type of foraging well, a ‘stopping rule’ should be implemented in order to try to understand whether people are making different decisions about when to stop for different conditions. We hope to extend the model in this way in the future. However, as you state, the model as it stands should be able to cope with non-exhaustive datasets (and in fact, to the best of our understanding, the Tagu & Kristjánsson 2021 value dataset is non-exhaustive, as participants did not have to find all 54 targets on each trial) and we have edited the discussion slightly to highlight this (lines 343-345).***

Specific comments:

- Figures: the figures often have multiple panels and which panel is presented in the text is not always very clear. Please consider labelling the panels with letters.

***We think this is mostly an issue with Figure 3, and we hope that adding labels here has helped make this more comprehensible.***

- l.8-9: a parenthesis seems to be missing here

***Thank you, this has been fixed.***

- l.223-227: could the authors indicate which target type is target 0? The same colours (red, green, yellow, blue) are often used by different foraging studies. Is the preference for target type 0 a reliable preference for a given colour (e.g. red) found in several datasets, or is it specific to the dataset that is analysed here?

***This was actually something we weren’t sure about from the original paper: it is not completely clear to us from the data file, as this column is simply labelled ‘target ID’. If we assume that the symbol condition column labels are in order, this corresponds to the ‘red square’ (in condition 3) and ‘green square’ (in condition 4) conditions, seemingly suggesting that participants had a preference to start with squares (possibly they seem slightly larger?) But we preferred not to say anything too specific here, as the experiment was clearly not designed to test this hypothesis and we wouldn’t want to generalise based on this posthoc analysis - but it would be an interesting question for future experiments.***

- l.261: typo, “figure figure 5”

***Thank you, this has been fixed.***

- l.271-272: I must be missing something here, but the text is referring to a negative direction bias while in figure 6 I (mistakenly?) see a positive direction bias.

***This plot indicates the direction weightings: on the x axis, we show the actual physical change in direction, so pi can be thought of as a complete reversal in direction, and 0 as carrying on in exactly the same direction. Thus, these plots show our model is giving greater weights to directions closer to pi, indicating that the participants are displaying negative momentum (more likely to double back on themselves). We have tried to make this interpretation clearer in the figure legend.***

- Supp. Materials part 1, Figure 9: as the curves are similar, it is difficult to identify the four distributions. Maybe using line colour instead of filling colour in the legend would help identify the curves on the plot. Same thing for Fig.3 of supplementary materials part 2.

***In Figure 9, the prior distributions do entirely overlap, which causes a bit of an issue! The figure caption now states this explicitly, and we have edited the line colours as suggested which we hope helps slightly with p_a_ and p_s_. We have also made similar alterations for Figure 3 in the supplementary materials part 2.***

- Supp. Materials part 1: a small bias on pS is tested with a preference of .6 (end of p. 8), while a small bias on pA is tested with a preference of .8 (p. 9). But is .8 really a “small” preference? Why not using a bias of .6 in both analyses?

***This has now been edited as suggested.***

- Supp. Materials part 1, end of p. 17: it is said that the experiment contains 20 trials per condition, and then that the analysis was restricted to trials 5-25. So there appears to be a typo somewhere here!

***Apologies, this should have read trials 6-25. This has now been corrected.***
